# ASAS-NANP symposium: mathematical modeling in animal nutrition: synthetic database generation for non-normal multivariate distributions: a rank-based method with application to ruminant methane emissions

**DOI:** 10.1093/jas/skaf136

**Published:** 2025-05-04

**Authors:** Luis O Tedeschi

**Affiliations:** Department of Animal Science, Texas A&M University, College Station, TX, USA

**Keywords:** correlation preservation, methane, multiple linear regression, non-normal distributions, random forest, synthetic data

## Abstract

This study addresses the challenge of limited data availability in animal science, particularly in modeling complex biological processes such as methane emissions from ruminants. We propose a novel rank-based method for generating synthetic databases with correlated non-normal multivariate distributions aimed at enhancing the accuracy and reliability of predictive modeling tools. Our rank-based approach involves a four-step process: 1) fitting distributions to variables using normal or best-fit non-normal distributions, 2) generating synthetic databases, 3) preserving relationships among variables using Spearman correlations, and 4) cleaning datasets to ensure biological plausibility. We compare this method with copula-based approaches to maintain a preestablished correlation structure. The rank-based method demonstrated superior performance in preserving original distribution moments (mean, variance, skewness, kurtosis) and correlation structures compared to copula-based methods. We generated two synthetic databases (normal and non-normal distributions) and applied random forest (**RF**) and multiple linear model (**LM**) regression analyses. RF regression outperformed LM in predicting methane emissions, showing higher *R*^2^ values (0.927 vs. 0.622) and lower standard errors. However, cross-testing revealed that RF regressions exhibit high specificity to distribution types, underperforming when applied to data with differing distributions. In contrast, LM regressions showed robustness across different distribution types. Our findings highlight the importance of understanding distributional assumptions in regression techniques when generating synthetic databases. The study also underscores the potential of synthetic data in augmenting limited samples, addressing class imbalances, and simulating rare scenarios. While our method effectively preserves descriptive statistical properties, we acknowledge the possibility of introducing artificial (unknown) relationships within subsets of the synthetic database. This research uncovered a practical solution for creating realistic, statistically sound datasets when original data is scarce or sensitive. Its application in predicting methane emissions demonstrates the potential to enhance modeling accuracy in animal science. Future research directions include integrating this approach with deep learning, exploring real-world applications, and developing adaptive machine-learning models for diverse data distributions.

## Teaser

This study introduces a novel rank-based method that generates synthetic agricultural data while preserving complex relationships and biological plausibility. Random forest regression outperforms linear regression in methane prediction but shows less robustness across distributions. The approach enhances modeling accuracy in data-scarce agricultural and environmental sciences.

## Introduction

The adaption of traditional models (i.e., mechanistic, concept-based) to fit within a data-driven artificial intelligence (**AI**) framework may not fully leverage the potential benefits of AI in agriculture (Tedeschi, 2019; Ellis et al., 2020). Instead, AI-oriented models should be developed independently to capture the unique data-driven insights they offer and subsequently integrated with mechanistic models, yielding hybrid, intelligent mechanistic models that describe the underlying principles of outcomes while handling massive amounts of data (Tedeschi, 2022b, 2023).

However, the vast amounts of curated data on which AI-oriented models rely, encompassing numerous variables and extensive records, may not always be available for specific needs and simulations, particularly in animal science production (Tedeschi, 2022b). This scarcity of big data is not unique to animal science. It is also prevalent in the medical field due to privacy regulations and the rarity of certain diseases (Abedi et al., 2022), in environmental science, where data quality, measurement, and accessibility pose significant challenges (Miller and Goodchild, 2015; Tedeschi et al., 2022), in building construction energy use (Baasch et al., 2021), and in economics due to privacy and proprietary data concerns (Einav and Levin, 2014), to name a few. Furthermore, the need for reliable and curated data, coupled with the challenges of proper data integration, adds another layer of complexity to an already strenuous situation. The limitations of real-world datasets necessitate innovative synthetic data generation methods across fields. These techniques aim to augment limited samples, safeguard sensitive information, rectify class imbalances, and simulate rare or hypothetical scenarios. Crucially, they strive to preserve the intricate relationships among variables, maintain statistical fidelity, and avoid introducing spurious correlations, thereby enhancing the robustness and applicability of data-driven models and analyses. In particular, the agricultural sector faces unique challenges in modeling complex biological processes, such as methane (**CH**_**4**_) emissions from ruminants, where comprehensive datasets are often limited given the difficulties associated with their measurement in the field (Tedeschi et al., 2022).

Although the definition of synthetic data continues to evolve, the concept of using computer-generated data to address specific tasks is well established. This approach traces back to the pioneering work of Stanislaw Ulam and John von Neumann in the 1940s, who developed Monte Carlo simulation methods to model complex systems during World War II (Metropolis and Ulam, 1949; Ulam, 1950; Beyer et al., 1985). Today, the landscape of synthetic data generation has expanded significantly, encompassing a wide array of advanced techniques. Deep learning (**DL**) architectures like generative adversarial networks (**GAN**) have revolutionized the production of highly realistic synthetic images, text, and medical data (Goodfellow et al., 2020). Variational autoencoders (**VAE**) provide a probabilistic approach to generating new data points with applications in image processing and anomaly detection (Kingma and Welling, 2019, 2022). In addition to these techniques, diffusion models have recently emerged as powerful tools for generating high-quality synthetic data, particularly in complex domains (Zhu, 2024). These models offer new possibilities for creating realistic datasets while preserving the statistical properties of the original data. Other methods, such as agent-based econometric models (Bonabeau, 2002) and stochastic differential equations used to simulate physical or economic systems (Carmona and Delarue, 2018a,b), also generate synthetic data. More recent advancements include the synthetic data vault (**SDV**), which uses probabilistic graphical models to generate multitable relational data (Patki et al., 2016), and differential privacy (**DP**) techniques that add controlled noise to preserve individual privacy while maintaining statistical utility (Dwork and Roth, 2014). Notably, Dwork et al. (2015) introduced the concept of the reusable holdout. This method allows multiple analyses on the same dataset while preventing overfitting and maintaining the validity of results, which is particularly valuable when working with limited or sensitive data. These sophisticated algorithms create realistic and high-quality datasets applicable across various fields, particularly medicine (Giuffrè and Shung, 2023). In finance, synthetic data plays a crucial role in overcoming data-sharing limitations due to regulatory requirements and privacy concerns. It can help simulate market conditions, test trading algorithms without financial risk, and enable cross-departmental collaboration within financial institutions. In crop science, Akkem et al. (2024) provided a comprehensive review of synthetic data generation in smart farming using GAN and VAE, highlighting the potential of these techniques in addressing data scarcity issues. As Assefa et al. (2021) highlighted, synthetic financial data generation faces unique challenges in creating realistic datasets, measuring similarities with actual data, and ensuring privacy compliance, all while navigating the complex regulatory landscape of the financial services industry. However, this approach not only facilitates internal data sharing but also opens up possibilities for broader research collaborations in the financial domain. For autonomous vehicle development, synthetic datasets are crucial in training and validating AI models, fostering safer and more efficient transportation solutions (Giuffrè and Shung, 2023). While these tools excel in image processing and data imputation, there remains a pressing need to develop datasets that accurately capture existing relationships among variables to serve diverse applications better, such as in environmental science, social policy modeling (Nikolenko, 2022), and agricultural sciences when data is limited.

Several methods have been developed to generate synthetic databases using correlated, non-normally distributed data, addressing the limitations of traditional approaches that often assume normality. Qu et al. (2020) described an approach based on the work of Vale and Maurelli (1983), which generates data based on the first four moments (means, variances, skewness, and kurtosis) while considering the correlation among the independent variables. This method extends the multivariate normal distribution to accommodate non-normal data. However, it requires detailed information on the four moments for each independent variable, which can be challenging when the variables have distinct distributions (e.g., triangular, log-logistic, β-general). Ruscio and Kaczetow (2008) proposed an iterative, trial-and-error process to sample from non-normal distributions while maintaining the desired correlation matrix to address these limitations. This approach offers more flexibility but can be computationally intensive for large datasets. Auerswald and Moshagen (2015) introduced a nonlinear structural equation modeling (**SEM**) approach that uses nonlinear linking functions and covariance corrections to generate synthetic data with specified moments and correlation structures, providing a more flexible and robust solution. More recent advancements include the work of Xu and Veeramachaneni (2018), who proposed a method using Gaussian copulas to model complex dependencies between variables while preserving their distributions. This approach can handle mixed data types and capture nonlinear relationships more effectively. Despite these advancements, there remains a need for approaches that effectively generate synthetic datasets by leveraging existing relational dependencies among variables, particularly when these variables deviate from the normal distribution assumptions. Furthermore, maintaining utility while ensuring privacy in synthetic data generation remains a critical challenge, especially in domains with sensitive information, such as health care and finance (Hittmeir et al., 2019; Murtaza et al., 2023). Such methods would not only preserve the inherent relationships among variables but also enhance the applicability and accuracy of synthetic datasets in various machine learning (**ML**) and simulation tasks.

The need for robust synthetic data generation methods is particularly evident in the field of agricultural science, where modeling complex biological processes often relies on limited datasets. A prime example of this challenge is in the study and prediction of CH_4_ emissions from ruminants. Methane emissions from ruminants are a significant contributor to greenhouse gases, impacting global climate change. According to Tedeschi and Beauchemin (2023), U.S. beef cattle alone emitted 22.6% of total agricultural emissions, representing about 2.2% of total anthropogenic emissions of CO_2_ equivalent. Developing accurate predictive models for CH_4_ production is crucial for devising effective mitigation strategies in animal agriculture. However, the complexity of the biological processes involved and the limitations in data collection (Hristov et al., 2018; Tedeschi et al., 2022) often result in datasets that are insufficient for modern AI modeling approaches.

The objective of this article is to address this challenge by proposing a robust synthetic data generation method that accommodates the complexities of multivariate non-normal distributions, which are commonly found in agricultural datasets but often neglected. We present a simple yet effective method for creating synthetic databases, enhancing the accuracy and reliability of predictive modeling tools such as random forest (**RF**) regression and simple linear multivariate regressions. To illustrate the practical application of this method, an existing CH_4_ production database from beef cattle is used as a case study, demonstrating how synthetic data can improve model predictions and provide deeper insights into CH_4_ emissions from ruminants. Tedeschi (2022a) and Tedeschi (2024) published a preliminary version of this study. Building upon this initial work, we have developed a web-based application of this technology at https://www.nutritionmodels.com/methane.html. This interactive web tool serves as a practical interface for researchers, nutritionists, and livestock managers to apply our CH_4_ prediction model to their specific scenarios.

## Materials and Methods

### Original database

**Figure 1.**
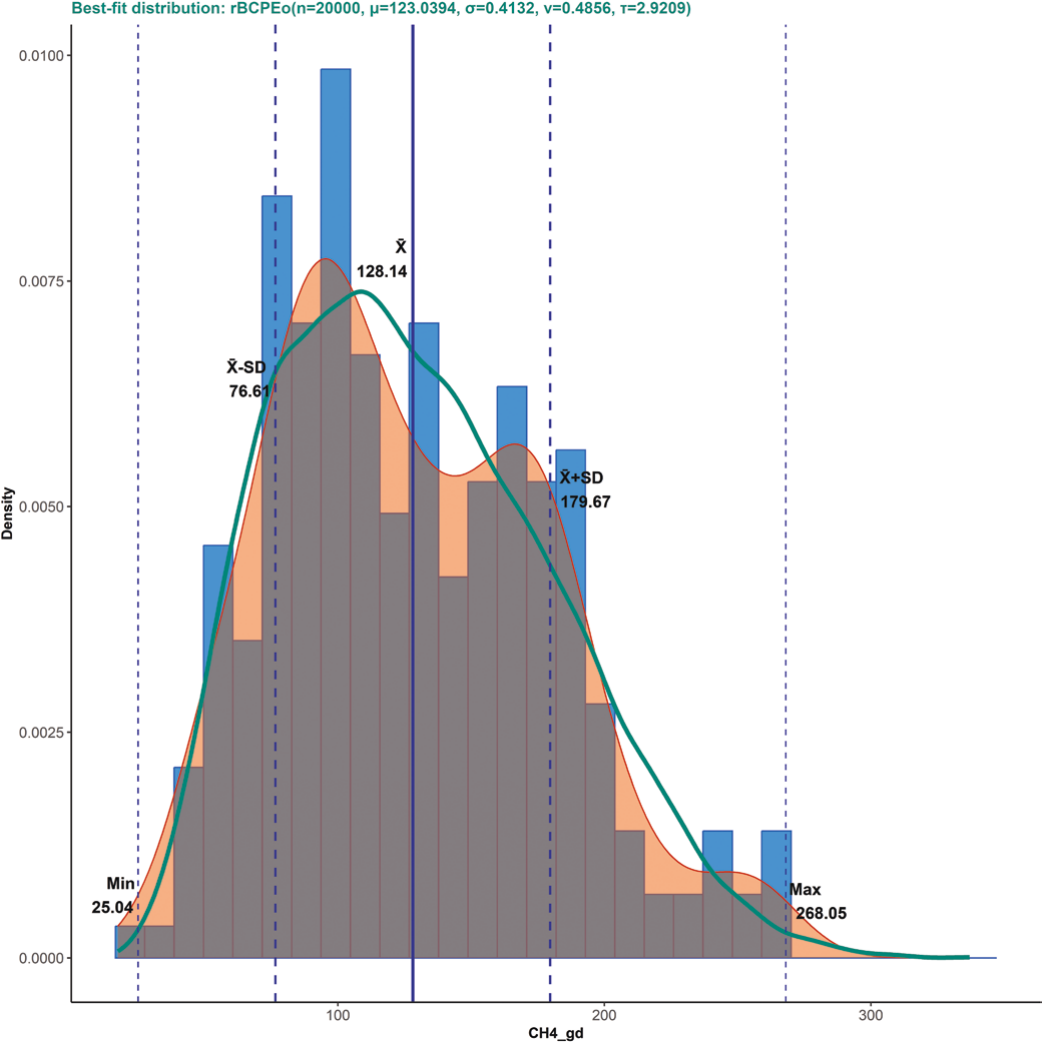
Histogram and density plots of methane (CH_4_, g/d) from the literature-gathered database. The blue bars represent the histogram, the orange shade indicates the density plot of the original data, the green line illustrates the density plot of the fitted data using the best-fit distribution shown at the top of the plot, and the vertical lines from left to right denote the minimum (min), mean—1 SD, mean, mean + 1 SD, and maximum (max) values in the literature-gathered database.

The original database was compiled from studies gathered by Galyean et al. (2016), supplemented with additional published research listed in Supplementary Table S1. The comprehensive dataset comprises 63 studies (34 initial and 29 additional), totaling 263 records. To ensure a generalized database with broad variability, reflecting the original non-normal distribution of the variables of interest, no specific selection criteria were applied. The dataset includes eight independent variables, mainly diet attributes, as follows: body weight (**BW**, kg), dry matter (**DM**) intake (**DMI**, kg/d), crude protein (**CP**, %DM), neutral detergent fiber (**NDF**, %DM), ether extract (**EE**, %DM), starch (%DM), acid detergent fiber (**ADF**, %DM), and ash (%DM), with CH_4_ production (g/d), or emission, as the dependent variable. Figure 1 has the histogram and density plots and best-fit nonlinear distribution for CH_4_, while Supplementary Figure S1 presents the same graphical information for the independent variables. It is crucial to note that the reported values are averages of measured values, representing estimated true population means. Consequently, the actual variability in the population may be greater than depicted, as the averaging process tends to smooth out extreme values and natural variations. This limitation should be considered when interpreting results and extrapolating findings to broader contexts.

### Synthetic databases

The *gamlss* package (Stasinopoulos et al., 2017; Rigby et al., 2019) of R v. 4.4.1 (R Core Team, 2019) was used to create synthetic databases. The process involved the following four steps. Step 1: For each variable in the dataset, distributions were fitted using either a normal distribution assumption or the best-fit distribution selected from over 100 different types available in the *fitDist* function of the *gamlss* package. It was implemented as shown in Supplementary Material S1. Step 2: This fitting process resulted in two synthetic databases, each containing 20,000 records. One database was generated using only normal distributions for each variable. The other database used the best-fit distributions for each variable, which could include normal distributions as well as other types. Step 3: Spearman correlations of the original database variables were calculated a priori using the *cor* function of R to preserve the relationships among variables. It was implemented as shown in Supplementary Material S2. Step 4: The synthetic datasets were cleaned to ensure consistency and biological plausibility based on the following criteria: 1) CP, EE, starch, NDF, ADF, and ash had to be greater than zero and less than 100; 2) ADF values had to be less than NDF values; and 3) the sum of CP, EE, ash, NDF, and starch had to be less than 100. The Spearman correlations from the original database variables were then applied to the cleaned synthetic datasets to ensure that the original variable relationships were maintained. These steps ensured that the synthetic datasets closely mirrored the characteristics of the original data while maintaining the necessary statistical relationships and realistic constraints.

### Correlation for non-normally distributed variables

The Cholesky decomposition, first introduced by André-Louis Cholesky in the early 20th century (Higham, 2002), is a mathematical technique primarily used for decomposing positive-definite matrices. It is commonly employed to generate correlated normal random variables in the context of multivariate normal distributions (Gentle, 2009). However, for non-normal distributions, the direct application of Cholesky decomposition alone is insufficient to maintain the desired correlation structure. This limitation arises because the decomposition assumes normality, and directly applying it to non-normal distributed variables may lead to distorted correlations. To address this issue and effectively generate correlated non-normal variables, two alternative methods were compared in this study.

#### Rank-based method

This method is based on four steps: normal data generation, correlation imposition, ranking maps, and allocation assignment. It starts by generating uncorrelated, normally distributed variables, and the Cholesky decomposition is applied to these variables to achieve the desired correlation. Then, the ranks of the values in each normally distributed variable are obtained. Finally, the original non-normally distributed variables are reorganized (i.e., sorted) according to the ranks of the corresponding normally distributed variables that have undergone the Cholesky decomposition. Supplementary Material S2 contains the R script code used to obtain the Cholesky decomposition for non-normally distributed variables in our simulations. This simple and computationally efficient approach aims to have non-normal variables retain their original distributions while achieving the desired correlation. However, there may be limited flexibility in controlling the degree of non-normality, and this method may not fully retain the original Spearman correlation. Additionally, it is necessary to investigate whether the Cholesky decomposition and reranking process accurately replicate the desired correlation and distribution moments, including mean, variance, skewness, and kurtosis.

#### Copula and SEM methods

Various methods exist to correlate multivariate non-normal distributions, including copulas and nonlinear SEM. The copula method, widely used for modeling dependencies between variables (Genest and MacKay, 1986; Nelsen, 2006), involves a multistep process: 1) generating synthetic data for each variable using best-fit non-normal distributions, 2) converting these to uniform distributions via the empirical cumulative distribution function, 3) transforming them to normally distributed marginals using the inverse normal cumulative distribution function, 4) adjusting the correlation structure iteratively to match the desired correlation matrix using Cholesky decomposition, 5) matching the ranks of the adjusted data to the ranks of the original non-normal data, and 6) iteratively checking to confirm convergence. This process aims to ensure that the data retains the original distributions while achieving the desired correlations. The nonlinear SEM method (Auerswald and Moshagen, 2015) generates synthetic data with specified moments using a different approach: 1) utilizing nonlinear linking functions and covariance corrections to adjust initial data, 2) achieving the desired correlation structure, including specific higher moments like skewness and kurtosis, and 3) iteratively adjusting to ensure the final synthetic data closely match the target distributions and correlations specified in the model. Both methods aim to preserve the complex relationships and distributions of the original data while generating synthetic datasets that accurately reflect these characteristics. For the sake of comparison with the rank-based method, Supplementary Material S2 shows the R script code of an application of the copula-based method.

#### Evaluation of distribution moments and correlations

To evaluate the preservation of the original non-normal distributions, including skewness and kurtosis, while imposing a desired correlation structure using the rank-based and copula methods, we generated synthetic databases comprising three variables, each following a distinct non-normal distribution (chi-square, beta, and log-normal), with 5,000 samples across 100 iterations. These distributions are independent of the process used to generate the synthetic databases, which contained 20,000 samples. The evaluation of distribution moments and correlations with 5,000 samples and 100 iterations was a separate analysis designed to assess the performance of the rank-based and copula methods under specific distributional assumptions (chi-square, beta, and log-normal). The 100 iterations refer to repeating this analysis to ensure the robustness of the correlation preservation method. Supplementary Material S3 has the R script code, showing a predefined positive-definite correlation matrix used for this simulation. Specifically, var1 was generated from a chi-square distribution with two degrees of freedom, var2 was generated from a beta distribution with shape parameters *a* = 2 and *b* = 5, and var3 was generated from a log-normal distribution with a mean of zero and a SD of one. Furthermore, we compared the preestablished Spearman correlation with the Spearman correlation of these distributions after reranking them using our method across all 100 iterations.

### Regression fitting, cross-testing, and adequacy

#### Predictive regressions

RF and multiple linear model (**LM**) regression analyses were performed on the synthetic databases to evaluate their predictive capabilities for CH_4_ emissions. The RF regression was implemented using the *randomForest* package (Breiman, 2001) in R v. 4.4.1 (R Core Team, 2019), with 150 trees and four randomly sampled variables as candidates at each split. This configuration was chosen based on preliminary analyses that showed optimal performance with these parameters. For the LM, we employed the ordinary least-squares method using the *lm* function in R v. 4.4.1 (R Core Team, 2019).

#### Cross-testing

To assess the robustness and generalizability of the models, we conducted a cross-testing analysis. This involved applying regressions developed on the normally distributed synthetic database to the non-normally distributed synthetic database and vice versa. This cross-testing approach allows for a more comprehensive evaluation of model performance across different data distributions.

#### Gini importance plots

To assess the relative importance of predictor variables in the RF model, we utilized two key indices: predictive accuracy score (**PAS**) and square error reduction (**SER**). The PAS measures a variable’s contribution to the model’s predictive accuracy. It is calculated as the percentage increase in mean squared error (**MSE**) when the variable’s values are randomly permuted. A higher PAS indicates that the variable is more crucial for maintaining the model’s predictive accuracy. The PAS is also known as the mean decrease in accuracy in classification tasks to measure how much the model’s accuracy decreases when a variable is excluded, with higher values indicating greater importance (Breiman, 2001). The SER is analogous to the mean decrease in Gini for classification tasks, and it quantifies the total decrease in node impurity from splits over a given variable, averaged across all trees. In regression contexts, SER (i.e., node impurity) is measured by the residual sum of squares, so its unit is the square unit of the dependent variable (i.e., daily CH_4_ emission, g^2^/d^2^). Higher SER values suggest a variable’s effectiveness in creating homogeneous groups (i.e., high precision) within the decision trees of the RF. These measures offer complementary insights: PAS focuses on predictive accuracy, while SER reflects the variable’s role in the tree structure (i.e., precision). Variables with greater SER values are considered more important in the model’s decision-making process, while variables with greater PAS values increase the accuracy of the predictions. Strobl et al. (2008) noted that correlations between predictor variables and differences in variable scales can influence these importance measures. The Gini importance plot displays the PAS and the SER values for each feature, and the variables are typically sorted in descending order of SER. This allows for a straightforward visual interpretation of feature importance, where features at the top of the plot are the most influential in the model’s predictions. However, it is essential to note that while Gini importance provides valuable insights, it can be biased towards high-cardinality features and does not indicate the direction (positive or negative) of a feature’s influence (Strobl et al., 2007). Therefore, we interpret these plots in conjunction with other model adequacy metrics (Tedeschi, 2006) to gain a comprehensive understanding of our RF model’s behavior and the underlying relationships in our data.

#### Prediction adequacy

The predictive accuracy of the CH_4_ emission models was evaluated using a suite of statistical measures, as recommended by Tedeschi (2006). These included mean bias (**MB**) to assess systematic over- or underprediction; mean square error of prediction (**MSEP**) and its root (**RMSEP**) to quantify overall prediction error; MSEP further decomposed into MB, slope bias (i.e., systematic deviation in the predicted slope from the true slope, indicating how well the model captures the relationship between input and output variables), and random errors (i.e., uncontrolled inherent variability or noise in the data) to provide insights into the sources of prediction error; concordance correlation coefficient and its accuracy estimate (**C_b_**) to evaluate the agreement between predicted and observed values; and Akaike’s Information Criterion (**AIC**) to compare model fit while accounting for model complexity. This comprehensive set of statistical measures provides a robust framework for assessing and comparing the performance of the RF and LM regressions across different synthetic data distributions.

## Results and Discussion

### Distribution moments and correlations

#### Distribution moments

Based on our evaluation of the distribution moments and correlation across the 100 iterations (Supplementary Material S3; data not shown) for the rank-based method, the mean of the original and correlated data was preserved for each distribution (chi-square, beta, and log-normal), indicating that the rank-based method effectively maintains the mean. The variance of the original and correlated data also remained consistent, showing that the method did not distort the spread of the data. The skewness values of the original and correlated data were very similar, indicating that the method preserves the asymmetry of the distributions. The kurtosis values of the original and correlated data matched closely, demonstrating that the method retains both the tail behavior and the central peak characteristics (i.e., peakedness) of the distributions. Over the 100 iterations, the differences between the original correlation (i.e., the preestablished matrix) and the effective correlation using the rank-based method varied from −0.0271 to 0.0428 for var1-var2, −0.174 to 0.0455 for var1-var3, and −0.0219 to 0.0419 for var2-var3. Our rank-based method (Supplementary Material S2) to correlate multivariate non-normal distributions effectively maintained the mean, variance, skewness, and kurtosis of the original non-normal distributions while imposing the desired correlation structure. Similarly, the copula-based approach (Supplementary Material S2) maintained the moments of the original and correlated data. However, the copula-based approach showed more variability and less precision in maintaining the desired Spearman correlations. The differences between the original correlation (i.e., the preestablished matrix) and the effective correlation using the copula-based method varied from −0.488 to 1 for var1-var2, −0.689 to 0.464 for var1-var3, and −0.599 to 0.357 for var2-var3. This method might be more suited for maintaining Pearson correlations than Spearman correlations, mainly when applied to non-normal data. The large discrepancies in the Spearman correlations between the original and correlated data are intriguing and cast doubt on the ability of copula-based methods to yield preestablished, correlated, non-normally distributed variables adequately. It is also likely that the implementation in Supplementary Material S2 needs further refinement. When using a variable correlation matrix instead of the preestablished matrix, the discrepancies increased further. This finding confirms that the rank-based method provides reasonable control over the higher moments of the distributions beyond just rank correlation.

#### Spearman correlation matrix

The original Spearman correlation matrix (COR1) of the literature-gathered database to predict CH_4_ emissions in beef cattle (*n* = 263 records; Supplementary Table S1) is shown in equation 1. Similarly, the Spearman correlation matrix (COR2) of the cleaned synthetic database (n = 9,484 after the removal of outliers and nonconforming data points) is shown in equation 2. The removal of outliers and nonconforming data points was based on biological plausibility criteria to ensure that the synthetic dataset adhered to realistic dietary constraints. Nonconforming data points included cases where the sum of dietary components exceeded 100% and values where ADF was higher than NDF, among others, as detailed earlier. Outliers were identified using a combination of statistical techniques, such as Z-scores and interquartile range (Tedeschi, 2022b), to flag data points that significantly deviated from the distribution of the original dataset. It is acknowledged that generating synthetic data requires simulating almost twice the amount of data to achieve a biologically plausible and usable dataset, which is a limitation of the approach. While this process ensured that the synthetic data maintained realistic constraints, it reflects the narrow range of parameters present in the original dataset of fewer than 300 data points. Therefore, the limitation is not only the small size of the real dataset but also its representativeness of a larger latent set of data. The actual data were not edited or modified beyond standard data cleaning steps, but the limited scope of the actual data highlights the need for expanding real-world datasets in future studies. The synthetic database was developed using the best-fit non-normal distributions (*getDist* shown in Supplementary Material S2) for each variable, and their values were ranked using the methodology outlined in Supplementary Material S2 (*correlate_using_ranks* function).


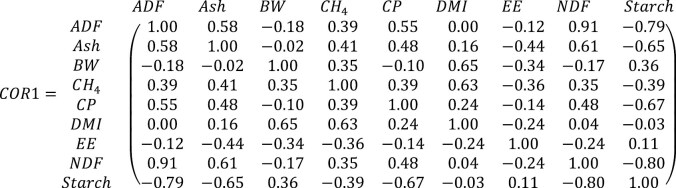
 (1)


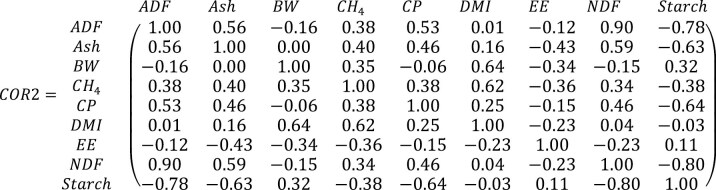
 (2)

where ADF is acid detergent fiber, % DM; Ash is % DM; BW is body weight, kg; CP is crude protein, % DM; DMI is DM intake, kg/d; EE is ether extract, % DM; NDF is neutral detergent fiber, % DM; and Starch is % DM.

As noted by Akkem et al. (2024), a fundamental challenge in generating synthetic crop data lies in accurately capturing the intricate temporal and spatial dependencies inherent in crop growth and environmental factors. This challenge underscores the critical importance of ensuring that the synthetic data generation process preserves existing relationships among variables while avoiding the introduction of spurious correlations or artificial patterns. In the context of generating synthetic databases, maintaining this delicate balance is crucial because the effectiveness of these systems relies heavily on the authenticity and representativeness of the underlying data. In some instances, the synthetic data retained small, nonzero correlations, for example, between certain diet attributes and BW. These nonzero correlations, however, are statistical artifacts necessary to maintain a stable and mathematically valid correlation matrix. Technically, these correlations are not significantly different from zero and are not biologically meaningful. The observed values reflect the structure of the original data, but the expected value for these relationships would be zero. As such, these artifacts should not impact the overall interpretation of the results or the predictive performance of the models. In our case, both matrices exhibit similar overall structures, indicating that the synthetic data generation method has successfully preserved the general pattern of correlations without adding patterns (i.e., correlation matrices are very similar). The majority of the differences between COR1 and COR2 are minor, suggesting that the method effectively maintains the original correlation structure. A few correlations exhibit moderate differences, such as those involving CP with BW, starch with NDF, and ash with BW. These differences, while noticeable, are not significant enough to drastically impact the overall distribution structure. Several correlations show negligible differences, suggesting that the synthetic data closely approximates the original data’s correlation structure in many instances. The minor and negligible differences indicate that the synthetic data generation method is robust and does not significantly alter the distribution’s structure. The preservation of correlation, along with mean, variance, skewness, and kurtosis, indicates that the synthetic data maintains the statistical properties of the original data. While moderate differences in some correlations might slightly affect specific relationships between variables, these are not widespread, and the overall impact on the distribution is likely minor.

When evaluating the Spearman correlations between the original and synthetic data generated over 100 iterations for chi-square, beta, and log-normal distributions (data not shown), we observed a difference of around 0.25 between the chi-square and beta distributions, while the differences between chi-square and log-normal, as well as beta and log-normal, were less than 0.1 for the rank-based method. The histograms of the differences in Spearman correlations between the original and correlated data are centered around zero, indicating that the rank-based method closely preserves the correlation structure. Conversely, the copula-based method showed more significant and inconsistent differences. The histograms of the differences in Spearman correlations for the copula-based method displayed more significant variability, indicating less precision in maintaining the desired correlation structure (data not shown).

However, it is crucial to note that our analyses, while comprehensive in examining overall statistical properties and correlations, do not preclude the possibility of inadvertently creating artificial relationships within subsets of the synthetic database. This limitation arises from the inherent complexity of multivariate datasets and the potential for subtle interactions that broader statistical measures may not capture. When working on modeling tabular data using Conditional GAN, Xu et al. (2019) highlighted the difficulties in generating synthetic data that faithfully preserved the statistical properties of the original dataset. Future work should involve more granular analyses of variable interactions, possibly employing techniques such as partial correlation analysis or advanced ML methods for detecting complex, nonlinear relationships. Additionally, field experts could provide valuable insights into the practical implications of any artificial relationships that may have been introduced during the synthetic data generation process.

### Predicting methane emissions

**Figure 2.**
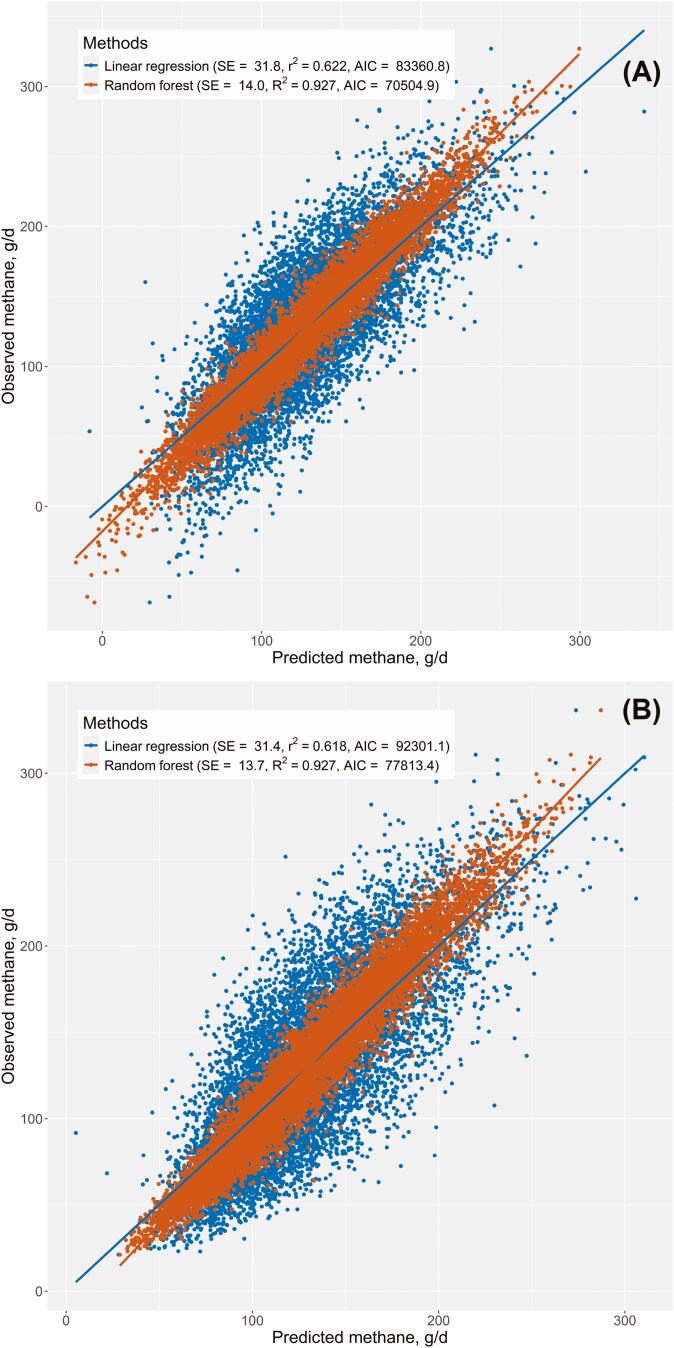
Relationship between observed (y-axis) and predicted (x-axis) methane (g/d) using multiple linear regression and RF regression, assuming (A) normal and (B) non-normal distributions of the independent variables.

Figure 2 shows scatter plots comparing the observed CH4 values from the synthetic dataset with the predicted CH_4_ emissions using LM and RF regressions. Figure 2A presents the results under the assumption that all key variables follow a normal distribution, while Figure 2B presents the results without this assumption. Note that some variables might follow a normal distribution naturally. The RF regression yielded a higher *R*^2^ (0.927) compared to the LM (0.622 and 0.618), regardless of the assumed distributions of the critical variables. Additionally, the RF regression had approximately half the SE of the LM regressions for both normal (Figure 2A) and non-normal (Figure 2B) distributions. The AIC was also lower for the RF than for the LM regressions. Table 1 provides further adequacy statistics, reinforcing the finding that the RF regression outperformed the LM regression in predicting CH_4_ emissions from the synthetic database. Because these are fitting regressions, one would expect the means of the observed and predicted independent variables (i.e., CH_4_) to be nearly identical, resulting in an MB close to zero, which is confirmed by the results (Table 1). However, the MSE for the RF regressions was approximately 19% of that for the LM regressions under both conditions (normal and non-normal distributions). Furthermore, while 100% of the MSE for the LM regressions was due to random errors, the RF regressions had about 19% of the MSE attributable to slope bias.

**Table 1.** Diverse fitting and predictive statistics of 2 × 2 combination for data generation and regression fitting using normal and non-normal distributions for LM or RF regressions

Items^1^	Normal distributions				Non-normal distributions			
	Normal regressions		Non-normal regressions		Normal regressions			Non-normal regressions
	RF	LM	RF	LM	RF	LM	RF	LM
*N*	8,542	8,542	8,542	8,542	9,484	9,484	9,484	9,484
Mean								
Predicted	128.5	128.6	129.7	128.5	126.4	129.6	128.5	128.4
Observed	128.6	128.6	128.6	128.6	128.4	128.4	128.4	128.4
MB	0.07	0.00	−1.05	0.11	1.91	−1.22	−0.17	0.00
SD								
Predicted	44.1	40.8	39.4	41.1	38.7	39.9	43.2	39.9
Observed	51.7	51.7	51.7	51.7	50.8	50.8	50.8	50.8
RMSE^2^	14.0	31.8	33.6	32.1	33.0	31.9	13.7	31.4
*r* ^2^	0.93	0.62	0.58	0.61	0.58	0.61	0.93	0.62
CCC	0.96	0.77	0.73	0.76	0.73	0.76	0.96	0.76
*C*_*b*_	0.99	0.97	0.96	0.97	0.96	0.97	0.99	0.97
MSE^2^	195.5	1,011.0	1,130.5	1,030.3	1,091.2	1,015.9	188.7	984.7
MB,%	0.0	0.0	0.1	0.0	0.3	0.1	0.0	0.0
Slope,%	18.9	0.0	0.0	0.0	0.0	0.0	19.3	0.0
Random,%	81.1	100.0	99.9	100.0	99.7	99.8	80.7	100.0
AIC	70,505	83,361	85,495	92,301	94,456	83,361	77,813	92,301

^1^AIC = Akaike’s Information Criterion, CCC = concordance correlation coefficient, *C*_*b*_ = accuracy, MB = mean bias, MSE = mean square error, *r*^2^ = coefficient of determination adjusted to number of variables, and RMSE = root of the mean square error.

^2^For statistical models with large databases, the MSEP and MSE tend to yield similar values as MSE = MSEP × n/(*n* − *p*); where *p* is the number of parameters. The ratio *n*/(*n* − p) is close to 1 for *n* = 10,000 and *p* = 9.

**Figure 3.**
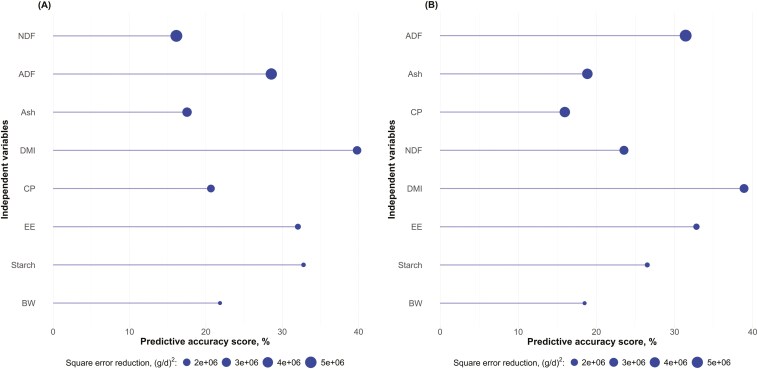
The Gini importance plot shows the increase in mean square error if the variable is removed from the model (PAS, %; line length) and how effectively a variable reduces residual square error when used for splitting in the RF’s decision trees (SER, (g/d)2; point size), assuming (A) normal and (B) non-normal distributions of the variables.

Figure 3 depicts the Gini importance plots for RF regressions under the assumption that all key variables follow a normal distribution (Figure 3A) or without this assumption (Figure 3B). In both distribution scenarios, DMI emerged as the most influential predictor based on PAS (normal: 39.80%, non-normal: 38.89%), meaning that randomly shuffling DMI values while keeping other variables unchanged increased the model’s MSE by 39.80% in the normal distribution scenario and 38.89% in the non-normal distribution scenario, decreasing the model’s accuracy. This result aligns with the findings by Hristov et al. (2018), who identified DMI as a primary driver of enteric CH_4_ emissions in cattle. The EE showed consistent importance across both distributions (normal: 32.05%, non-normal: 32.80%), supporting Grainger and Beauchemin’s (2011) observations on the impact of dietary lipids on CH_4_ production. Interestingly, the importance of fiber components differed between the two distributions. In the non-normal distribution model, ADF showed increased importance (PAS: 31.42% vs. 28.56% in the normal distribution), while NDF importance also increased (PAS: 23.53% vs. 16.13%). This shift suggests that accounting for non-normality may better capture the complex relationship between fiber content and CH4 emissions. This finding aligns with those from Appuhamy et al. (2016), who noted that dietary NDF concentration was one of the most frequently appearing feed variables in evaluated CH4 prediction models. Van Soest (1994) also emphasized the critical role of fiber in ruminant nutrition and its impact on CH_4_ production. The SER values provide a complementary perspective. In both distributions, NDF and ADF showed the highest SER values, underscoring the critical role of fiber in determining node purity in the RF model. However, the relative importance of these variables shifted, with ADF showing higher SER in the non-normal model (5,733,672 vs. 4,770,052 in normal), while NDF’s SER decreased (3,023,372 vs. 5,381,872). The CP and starch showed reduced importance in the non-normal model based on PAS (CP: 15.96% vs. 20.66%; starch: 26.51% vs. 32.79%). This suggests that assuming normality might overestimate the impact of these nutrients on CH4 emissions. These findings align with Dijkstra et al. (2011), who noted that increasing dietary CP content generally has little effect on CH4 production. The variable importance of starch across our models suggests a complex relationship with CH_4_ emissions. While Hatew et al. (2015) reported that the effect of starch source on CH_4_ production was not consistent, our results indicate that the importance of starch may be overestimated when assuming normal distribution. The BW maintained relatively consistent importance across both models in terms of PAS (normal: 21.85%, non-normal: 18.49%), supporting its inclusion as a stable predictor in CH_4_ emission models (Moraes et al., 2014), likely because the relationship between DMI and BW is represented by a linear relationship (i.e., power of 1) (Van Soest, 1994) though Ketelaars and Tolkamp (1992) showed evidence that DMI increases proportionally to the maintenance requirement (i.e., to the metabolic BW; power of 0.75).

The discrepancies in variable importance between the two distribution assumptions highlight the sensitivity of RF models to underlying data distributions. This underscores the importance of carefully considering data distribution when interpreting model results, particularly in complex biological systems like ruminant CH_4_ production. Our findings suggest that models assuming non-normal distributions may provide a more nuanced understanding of the factors influencing CH4 emissions, especially concerning dietary fiber content. These results emphasize the critical need to consider data distribution when developing predictive models for CH4 emissions. Assuming normality may lead to misinterpretation of the relative importance of specific dietary components, potentially impacting the accuracy of CH4 prediction models and subsequent mitigation strategies in ruminant production systems.

#### Cross-testing analysis

Table 1 also has the adequacy statistics for the cross-testing analysis. In this analysis, LM and RF regressions developed under non-normal distribution conditions were used to predict CH_4_ using a synthetic database created under normal distribution conditions of the independent and dependent variables, and vice versa. In the first scenario (non-normal regressions with normal distribution database), the RF regression underperformed compared to the LM regression. Although the MSE was similar between the RF (1130.5) and LM (1030.3) regressions, the RF regression had an MB of −1.05 g/d compared to 0.11 g/d for the LM regression. Similarly, in the second scenario (normal regressions with non-normal distribution database), the MSE was similar between RF (1091.2) and LM (1015.9) regressions, but the MB was greater for the RF (1.91 g/d) than for the LM (−1.22 g/d) regressions. Notably, there was a sign exchange in the MB during these cross-testing analyses. The cross-testing results in Table 1 suggest that the adequacy statistics for the LM regressions are independent of the nature of the distribution of the independent and dependent variables. In contrast, the RF regressions exhibit high specificity: RF regressions developed using normal distribution are only suitable for normal distribution predictions, and those developed using non-normal distributions are only suitable for predictions with synthetic databases generated from non-normal distributions of the independent and dependent variables. The LM regressions appeared to be robust to the distribution type of the synthetic database used for both development and evaluation purposes. Conversely, the RF regressions underperformed (as indicated by MB values far from zero, higher MSE, higher AIC, and lower *R*^2^; Table 1) when the distribution type of the development and evaluation of synthetic databases differed.

These findings highlight the importance of understanding the distributional assumptions behind different regression techniques when generating synthetic databases. Multiple linear regression models showed robustness across different distribution types, making them a more flexible choice when the distribution of data may vary. In contrast, RF regressions, while generally more accurate within the same distribution type, exhibited significant performance drops when applied to data with differing distributions. Various techniques exist for generating synthetic databases, each with its strengths and limitations. Methods such as GAN (Goodfellow et al., 2020) and VAE (Kingma and Welling, 2022) have gained popularity for creating high-fidelity synthetic data, particularly in fields like image processing and anomaly detection.

These DL approaches have shown a remarkable ability to capture complex data distributions and generate realistic synthetic samples. For instance, Esteban et al. (2017) demonstrated the use of GAN in generating synthetic electronic health records that preserved the statistical properties of the original data while ensuring patient privacy. However, these methods often require large amounts of data and computational resources, which might not be practical for all applications, especially in fields with limited data availability, such as animal science.

Another common approach involves copula-based methods, which can model dependencies between variables without assuming a specific marginal distribution (Nelsen, 2006). This approach can be advantageous when dealing with non-normal data because it provides flexibility in maintaining the original data structure. Patki et al. (2016) utilized copula-based methods in their SDV framework, demonstrating its effectiveness in generating synthetic relational databases that preserve statistical properties and relationships across multiple tables. However, the complexity of copula models and the computational burden they impose can be challenging to manage, especially for high-dimensional datasets. Furthermore, our preliminary analysis of the copula-based method (Supplementary Material S2) showed that it was not as good as other methods. It had more variability in preserving the original distribution moments compared to the rank-based method. The histograms of the differences in Spearman correlations between the original and correlated data for the copula-based method showed more spread, indicating less precision in maintaining the desired correlation structure. Finally, though the copula method provides flexibility in modeling dependencies between variables, it is more complex and computationally intensive than the rank-based method. Interestingly and somewhat unexpectedly, the copula-based method did not perform as well as the rank-based method in terms of preserving the correlations of the original data, suggesting that while the copula-based approach is theoretically robust and flexible, in practice, it may introduce more variability and requires substantial computational resources, making the rank-based method a more efficient and reliable choice for applications with constraints similar to ours.

In our study, the rank-based method used to generate synthetic data ensured that the non-normal characteristics of the original data were preserved. This method demonstrated a balance between simplicity and effectiveness, especially when the goal is to maintain the relational structure of the variables. Ruscio and Kaczetow (2008) proposed a similar approach, with an iterative algorithm for generating multivariate, non-normal data with specified intercorrelations, which has shown good performance in preserving both univariate distributions and correlations. The performance of RF regressions, although highly specific to the distribution type, underscores the potential need for hybrid approaches that combine the robustness of LM with the precision of RF under specific conditions. This finding aligns with recent trends in ensemble methods and hybrid models. For example, Zhang and Mahadevan (2019) proposed a hybrid approach combining copula-based modeling with ML techniques for uncertainty quantification in engineering applications, demonstrating improved performance over traditional methods.

Future research could explore integrating these techniques to leverage their respective strengths, potentially developing more versatile models capable of handling diverse data distributions. For instance, combining the rank-based method with elements of DL architectures could potentially yield a more robust and flexible synthetic data generation framework. Additionally, investigating the application of these methods in real-world scenarios beyond synthetic datasets would provide deeper insights into their practical utility and limitations. Furthermore, the field of DP (Dwork and Roth, 2014) offers promising avenues for generating synthetic data that not only preserves statistical properties but also provides strong privacy guarantees. Integrating DP techniques with our rank-based method could enhance the utility of synthetic data for sensitive applications, such as those involving medical or financial data. Lastly, the specificity of RF regressions to distribution types observed in our study suggests a need for adaptive ML models that can automatically adjust to different data distributions. Recent advancements in transfer learning and domain adaptation techniques (Wang and Deng, 2018) could potentially be applied to develop more flexible RF models that maintain high performance across varying data distributions.

### Considerations of sample size on overfitting and overconfidence

Another important consideration is the sample size of the synthetic database. We initially generated a 20,000-record synthetic database but ended up with approximately 10,000 usable records (Table 1) after removing spurious and nonbiological data that were generated randomly. This reduction occurred due to the strict biological constraints we imposed to ensure the synthetic data accurately reflected real-world scenarios. For instance, we removed records where the sum of dietary components exceeded 100% or where ADF values were higher than NDF values, which are technically possible in real life due to limitations in the methodology of these fiber components (Van Soest, 1994; Tedeschi and Fox, 2020) but theoretically and biologically flawed. The use of percentage-based dietary features (e.g., nutrient components summing to 100%) might have notably contributed to this data reduction, as these features impose strict biological limits on the synthetic data. While such reductions might not constantly occur in other datasets or contexts, they were necessary here to maintain the biological plausibility of the synthetic data. The final sample size of around 10,000 records is particularly relevant in the context of our regression analyses. For RF models, this sample size is generally considered sufficient to achieve stable and reliable results (Oshiro et al., 2012; Probst and Boulesteix, 2017). Oshiro et al. (2012) found that the performance of RF models tends to stabilize after about 128 trees, and our use of 150 trees aligns well with this finding. For the LM, the sample size is more than adequate, as the rule of thumb suggests a minimum of 10–20 observations per predictor variable (Toutenburg and Shalabh, 2009; Harrell, 2015). Our cross-testing approach, where we applied models developed on normally distributed data to non-normally distributed data and vice versa, further validates the robustness of our sample size. This method allows us to assess how well the models generalize across different data distributions, which is crucial given the potential variability in real-world data. While the reduction in sample size from 20,000 to 10,000 records might seem substantial, it actually demonstrates the rigor of our data-cleaning process (Tedeschi, 2022b) and ensures that our synthetic data closely mimics the biological constraints of real-world systems.

On the other hand, a point of concern is that a large sample size (i.e., 10,000 data points) used in our synthetic database for both LM and RF regressions can provide robust statistical power, but it may also lead to potential issues in model interpretation and performance evaluation. In the context of linear regression, it can lead to an inflation of goodness-of-fit measures and potentially misleading interpretations of model performance. As sample size increases, even minor effects can become statistically significant, potentially leading to overinterpretation of weak relationships (Lin et al., 2013; Wasserstein and Lazar, 2016). Similarly, as sample size increases, the coefficient of determination (*R*^2^ or *r*^2^) tends to stabilize around the population value; it may appear impressively high even when the predictive power of the model is limited (Malek et al., 2007). This phenomenon is particularly relevant in our study, where the LM regression showed consistent performance across different data distributions. For large samples, traditional goodness-of-fit measures may become less informative, and alternative metrics focusing on predictive performance should be considered, even when considering the regressor variables as random (Buja et al., 2019).

To prevent overfitting issues (Hawkins, 2004) in large databases, future evaluations should focus on the effect sizes and practical significance rather than solely on statistical significance (Sullivan and Feinn, 2012); adopt the use of adjusted *R*^2^ or other less sensitive sample-size metrics (Spiess and Neumeyer, 2010), such as the robust *R*^2^ (Tedeschi, 2006); and consider the use of regularization techniques (Friedman et al., 2010), such as lasso and ridge regressions. Similarly, for RF models, the risk of overfitting with large datasets is a significant concern. While RF is generally robust against overfitting due to its ensemble nature, the use of a large synthetic dataset may still lead to models that capture noise rather than true underlying patterns. Oshiro et al. (2012) reported that while increasing the number of trees would generally improve performance, there is a point of diminishing returns, typically around 128 trees. In our study, the high *R*^2^ values (0.927) observed for RF models, while indicative of good performance, could potentially be a result of overfitting to the synthetic data. Overfitting in RF might be mitigated by utilizing pruning techniques to reduce complexity in individual trees (Breiman et al., 1984) or employing k-fold cross-validation or out-of-bag error estimates to provide a more realistic assessment of model performance (Breiman, 2001).

In fact, adding more data may backfire because RF may make incorrect predictions with a high degree of confidence (i.e., high *R*^2^), mainly if the distribution structure is different from that used to generate the RF model, stemming from model uncertainty or calibration issues. This phenomenon is rooted in the nature of ensemble methods like RF, which can lead to overfitting and overconfidence in predictions (Grushka-Cockayne et al., 2016), especially when faced with out-of-distribution data. The issue of model calibration in ML, including RF, has been extensively studied. While RF regression is generally well-calibrated for in-distribution data, it can become miscalibrated when faced with data from different distributions (Niculescu-Mizil and Caruana, 2005). Furthermore, Kull et al. (2017) proposed methods to improve the calibration of RF regressions, acknowledging that they can become overconfident if not calibrated adequately. Their work highlights the need for careful consideration of model calibration, especially when working with large datasets or when applying models to data with potentially different distributions. The high *R*^2^ values observed in our study, despite poor generalization in cross-testing, exemplify this issue. As Probst and Boulesteix (2017) point out, “the out-of-bag error estimate, commonly used in Random Forests, can be overly optimistic,” which may contribute to overconfidence in model performance.

It is well established that some AI methods, specifically supervised ML, heavily depend on the quality of data used to train their structures, and ill-conditioned data inevitably leads to biased ML predictions. While the need for substantial data in ML is undeniable, we must be cautious of falling into the lack-of-data trap when attempting to increase the predictability of regression models. Although ML requires big data and big data often necessitates ML for analysis, the failures in ML predictions cannot be attributed solely to data scarcity, nor can they always be solved by simply demanding more data. This mutual dependency between AI and big data is not inconsequential. It may lead to a self-reinforcing cycle without a clear resolution, potentially resulting in a death spiral of ever-increasing data demands without proportional improvements in prediction accuracy (Tedeschi, 2022b). Our findings emphasize the critical importance of implementing robust validation techniques and carefully interpreting model performance metrics. This is particularly crucial when working with large synthetic datasets, which may not fully capture the complexity and variability inherent in real-world data distributions. The challenge lies not just in acquiring more data but in ensuring that the data—whether natural or synthetic—accurately represents the underlying phenomena we aim to model.

## Conclusion

The comparison between the rank-based method and the copula-based method for generating synthetic datasets revealed that the rank-based method more effectively preserved the original distribution moments (mean, variance, skewness, and kurtosis) and the correlation structure. The rank-based method was more straightforward to implement and provided more consistent results, making it a robust choice for maintaining relational dependencies in synthetic datasets. Conversely, the copula-based method, while flexible and capable of modeling complex dependencies, showed more variability in preserving the original data’s characteristics and was more computationally intensive.

The differences between the original (COR1) and synthetic (COR2) correlation matrices were mostly minor, with a few moderate discrepancies. These differences are not significant enough to drastically impact the overall distribution structure. The synthetic data generation method effectively maintains the correlation structure and distribution moments, making it a reliable approach for creating synthetic datasets with similar statistical properties to the original data. However, our analyses do not guarantee that artificial relationships between subsets of variables within the synthetic database have not been introduced. These potential artificial relationships could affect specific downstream analyses, and further scrutiny may be necessary to ensure the integrity of the synthetic data for specific applications. Our analyses also indicated that the RF regression consistently outperformed the LM regression with higher precision values, lower SE, and lower AIC values, regardless of whether the variables followed normal or non-normal distributions. However, while both LM and RF regressions perform well when the synthetic database and the prediction model share the same distributional assumptions, the LM regressions exhibit consistent predictability across different distribution types.

In contrast, the RF regressions demonstrate high specificity and significant performance degradation in cross-testing scenarios where the distributional assumptions differ. Furthermore, while key predictors like DMI and EE remain consistently critical in predicting CH_4_, the assumption of non-normal distribution reveals the presence of nuances in the relationship between dietary components (particularly fiber) and CH_4_ emissions. This finding highlights the need for distribution-aware modeling approaches in animal science, especially animal nutrition, to ensure more accurate and robust predictions of CH_4_ emissions from ruminants. This study provides insights into the comparative performance of RF and LM; other techniques from the broader spectrum of ML methods, such as ensemble boosting, artificial neural networks, or support vector machines, warrant further exploration.

## Supplementary Material

skaf136_suppl_Supplementary_Figure_S1

skaf136_suppl_Supplementary_Material_S1

skaf136_suppl_Supplementary_Material_S2

skaf136_suppl_Supplementary_Material_S3

skaf136_suppl_Supplementary_Table_S1

## Abbreviations

ADF: acid detergent fiber
AI: artificial intelligence
AIC: Akaike’s Information Criterion
BW: body weight
CCC: concordance correlation coefficient
C_b_: accuracy
CH_4_: methane
CP: crude protein
COR1: correlation matrix of the original database
COR2: correlation matrix of the synthetic database
DL: deep learning
DM: dry matter
DMI: dry matter intake
DP: differential privacy
EE: ether extract
GAN: generative adversarial networks
LM: linear model
MB: mean bias
ML: machine learning
MSE: mean square error
MSEP: mean square error of prediction
NDF: neutral detergent fiber
PAS: predictive accuracy score
RF: random forest
RMSEP: root of MSEP
SDV: synthetic data vault
SEM: structural equation modeling
SER: square error reduction
VAE: variational autoencoders
