# Supplementary material for: ASAS-NANP symposium: mathematical modeling in animal nutrition: synthetic database generation for non-normal multivariate distributions: a rank-based method with application to ruminant methane emissions
Source: J Anim Sci. 2025 May 4;103:skaf136. doi: 10.1093/jas/skaf136 (PMC12351256; doi:10.1093/jas/skaf136)

ASAS-NANP SYMPOSIUM: MATHEMATICAL MODELING IN ANIMAL NUTRITION: Synthetic Database Generation for Non-Normal Multivariate Distributions: A Rank-Based Method with Application to Ruminant Methane Emissions (**Supplementary Figure 1**)

Supplementary Figure 1. Histogram and density plots for acid detergent fiber (ADF, % DM), ash, body weight (BW, kg), crude protein (CP, % DM), dry matter intake (DMI, kg/d), ether extract (EE, % DM), neutral detergent fiber (NDF, % DM), and starch (% DM) from the literature-gathered database. The blue bars represent the histogram, the orange shade indicates the density plot of the original data, the green line illustrates the density plot of the fitted data using the best-fit distribution shown at the top of the plot, and the vertical lines from left to right denote the minimum (min), mean – 1 SD, mean, mean + 1 SD, and maximum (max) values in the literature-gathered database.

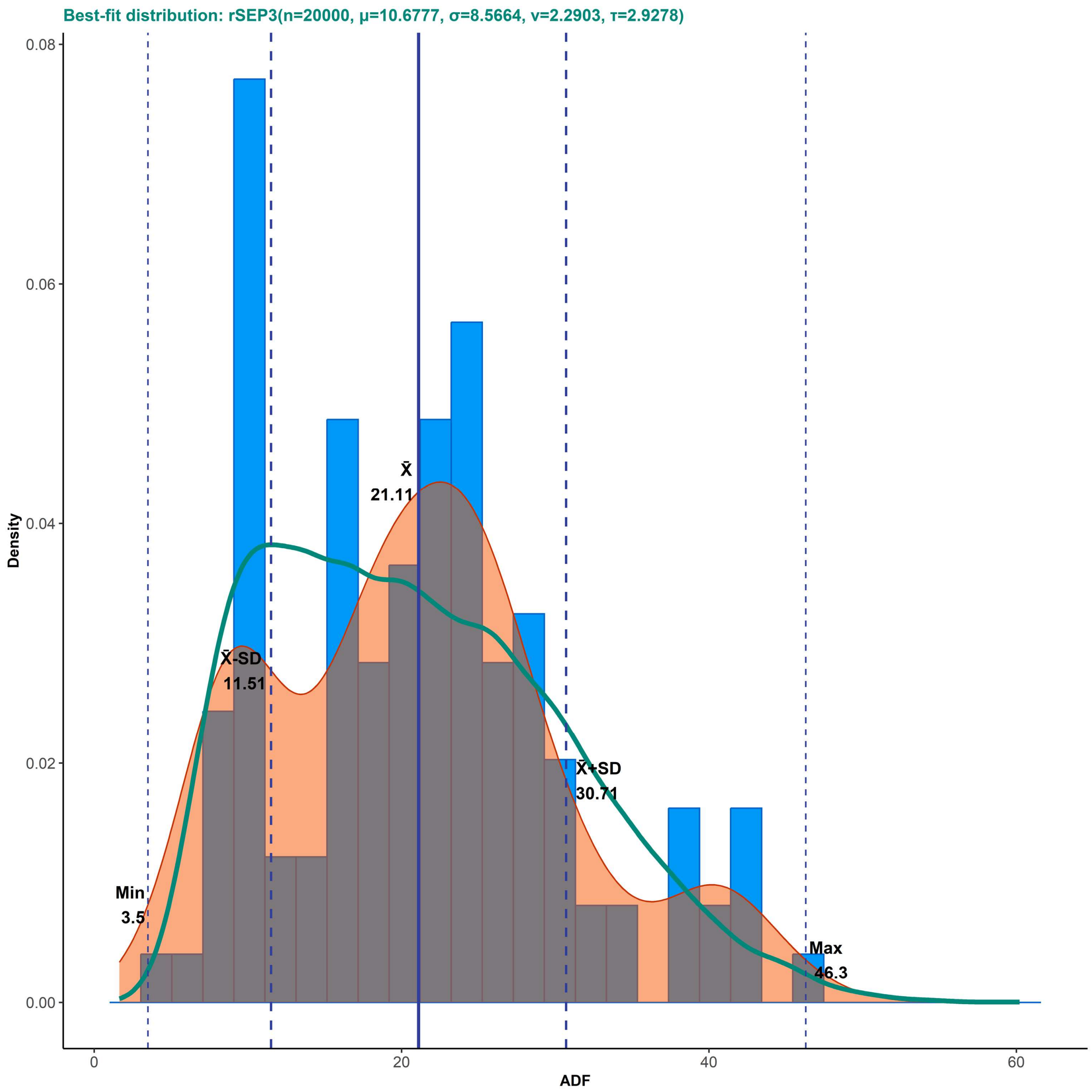

ASAS-NANP SYMPOSIUM: MATHEMATICAL MODELING IN ANIMAL NUTRITION: Synthetic Database Generation for Non-Normal Multivariate Distributions: A Rank-Based Method with Application to Ruminant Methane Emissions (**Supplementary Figure 1**)

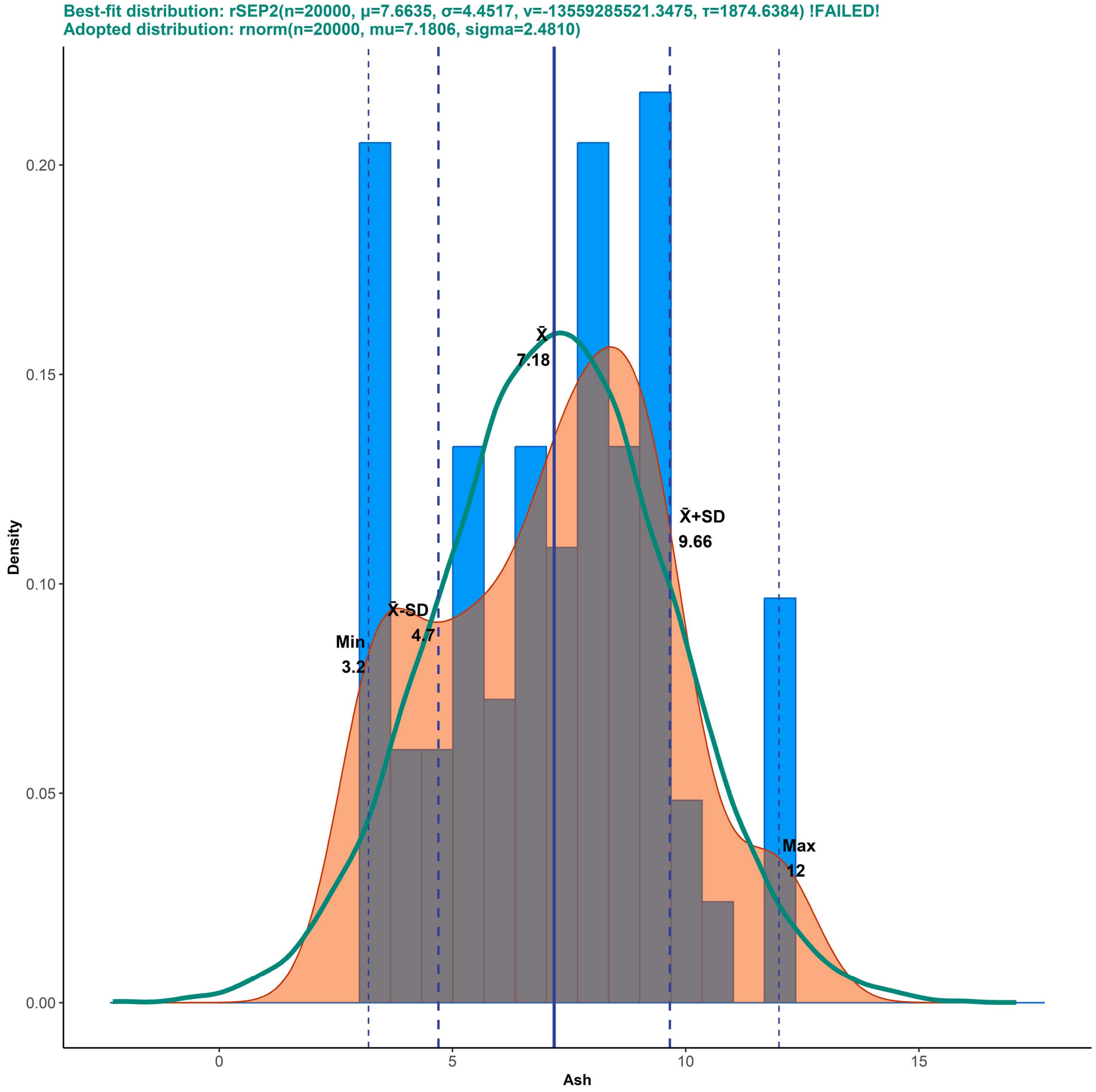

ASAS-NANP SYMPOSIUM: MATHEMATICAL MODELING IN ANIMAL NUTRITION: Synthetic Database Generation for Non-Normal Multivariate Distributions: A Rank-Based Method with Application to Ruminant Methane Emissions (**Supplementary Figure 1**)

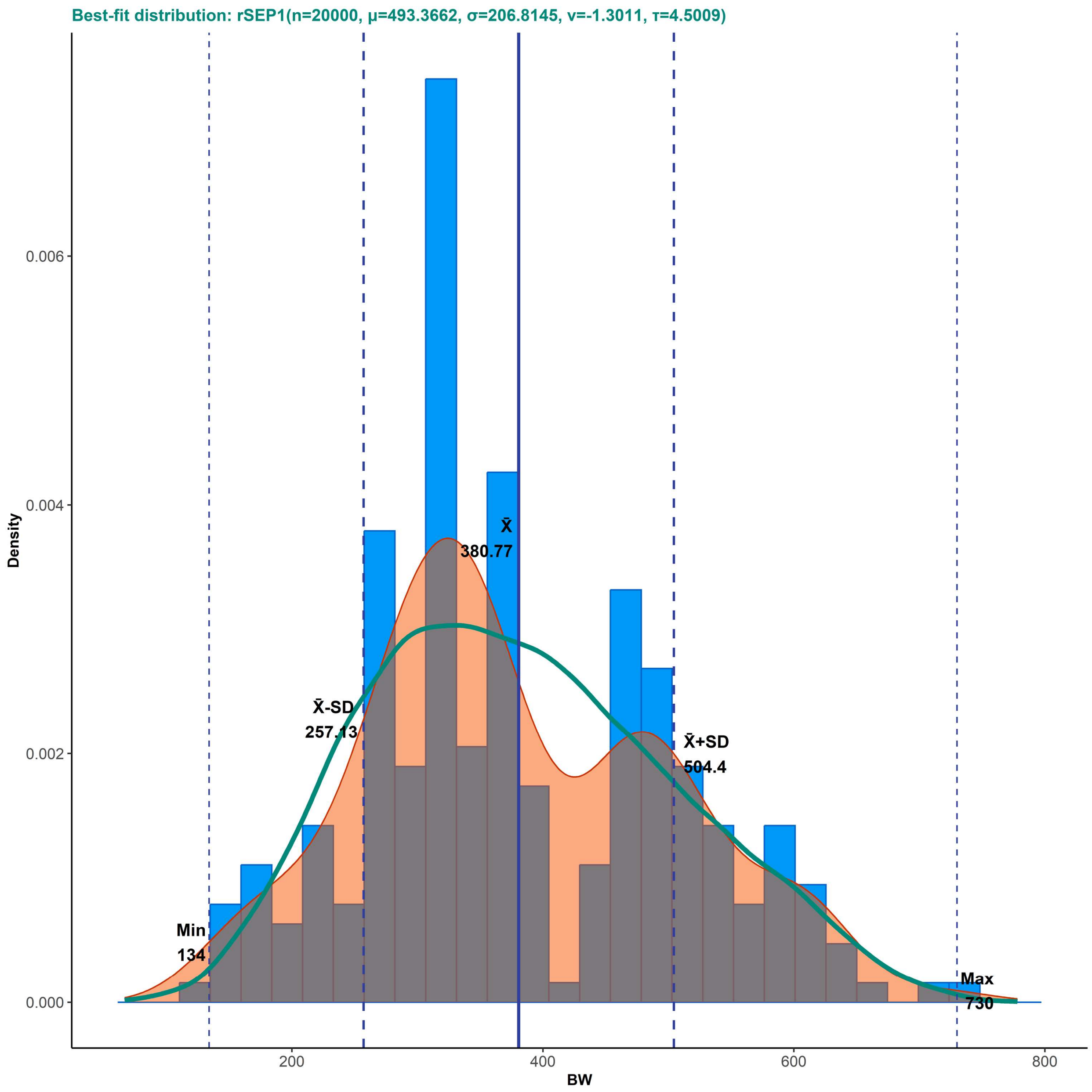

ASAS-NANP SYMPOSIUM: MATHEMATICAL MODELING IN ANIMAL NUTRITION: Synthetic Database Generation for Non-Normal Multivariate Distributions: A Rank-Based Method with Application to Ruminant Methane Emissions (**Supplementary Figure 1**)

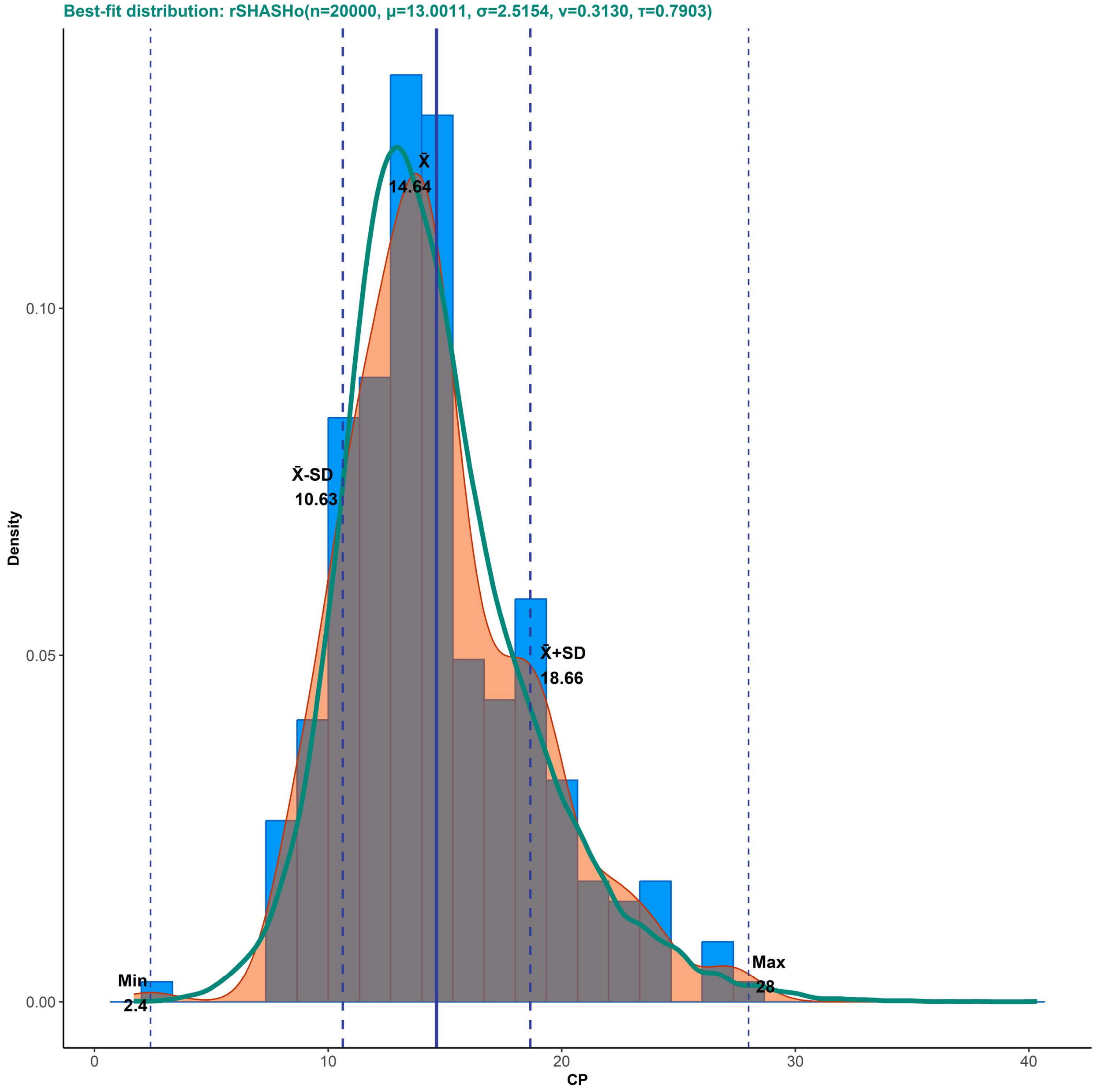

ASAS-NANP SYMPOSIUM: MATHEMATICAL MODELING IN ANIMAL NUTRITION: Synthetic Database Generation for Non-Normal Multivariate Distributions: A Rank-Based Method with Application to Ruminant Methane Emissions (**Supplementary Figure 1**)

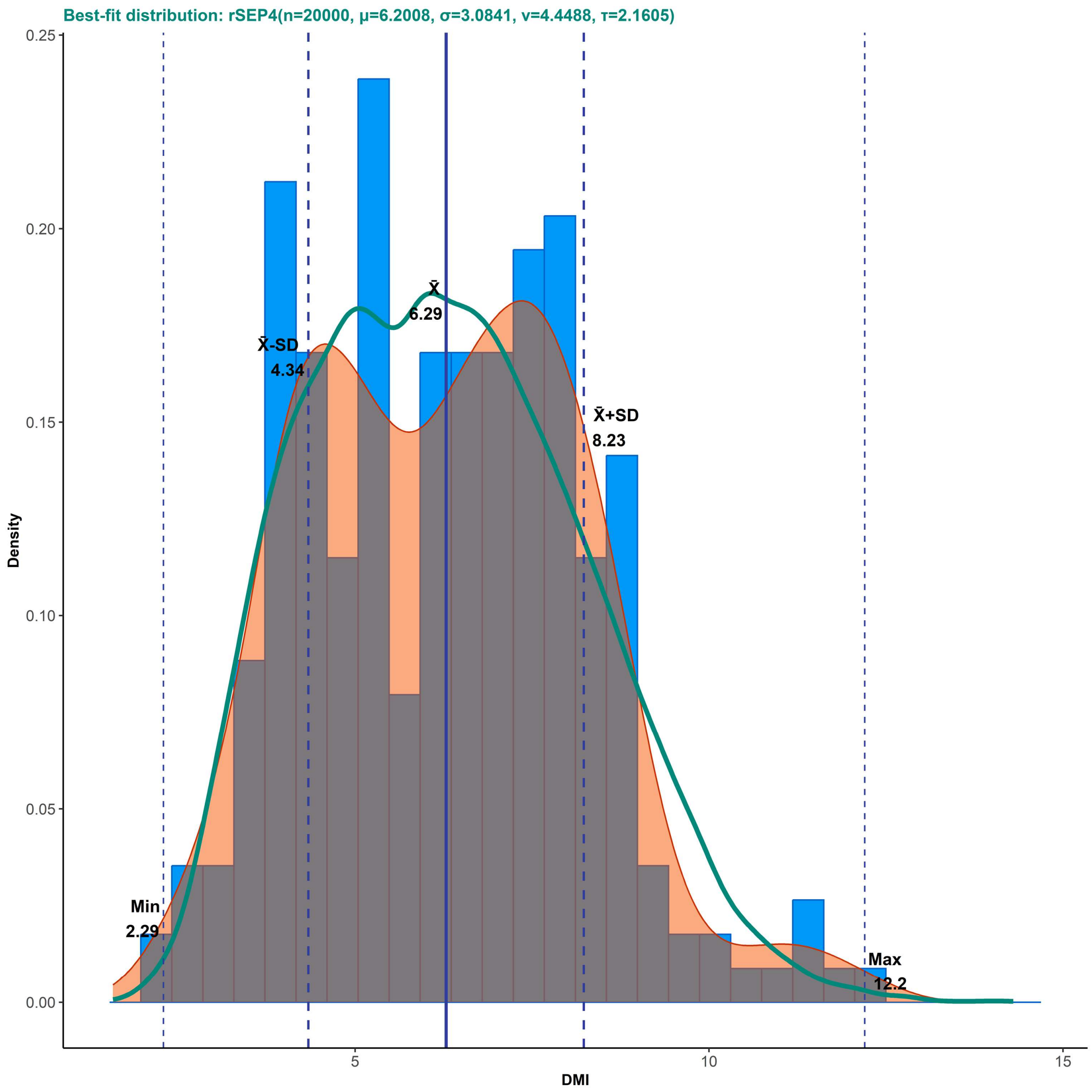

ASAS-NANP SYMPOSIUM: MATHEMATICAL MODELING IN ANIMAL NUTRITION: Synthetic Database Generation for Non-Normal Multivariate Distributions: A Rank-Based Method with Application to Ruminant Methane Emissions (**Supplementary Figure 1**)

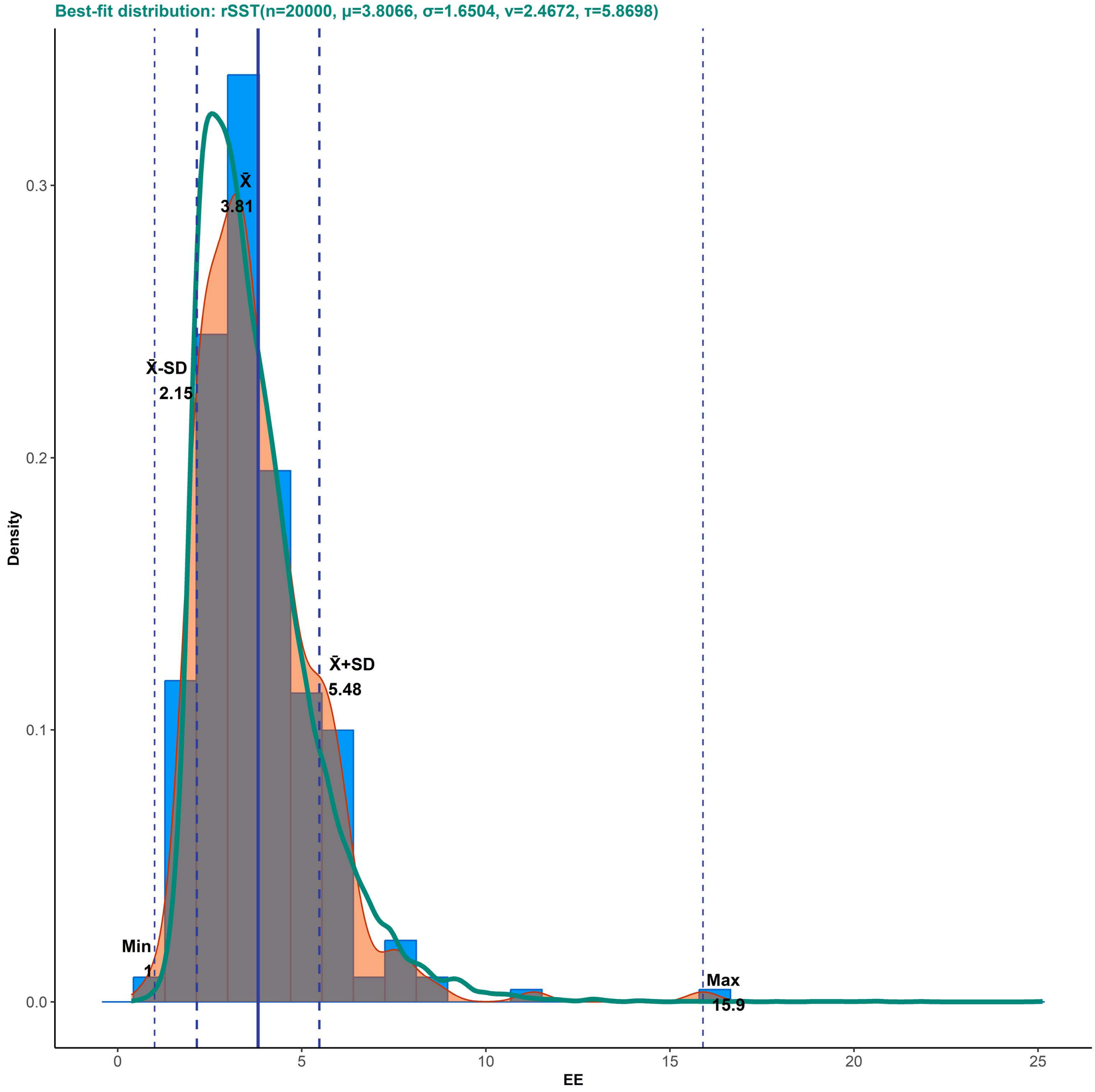

ASAS-NANP SYMPOSIUM: MATHEMATICAL MODELING IN ANIMAL NUTRITION: Synthetic Database Generation for Non-Normal Multivariate Distributions: A Rank-Based Method with Application to Ruminant Methane Emissions (**Supplementary Figure 1**)

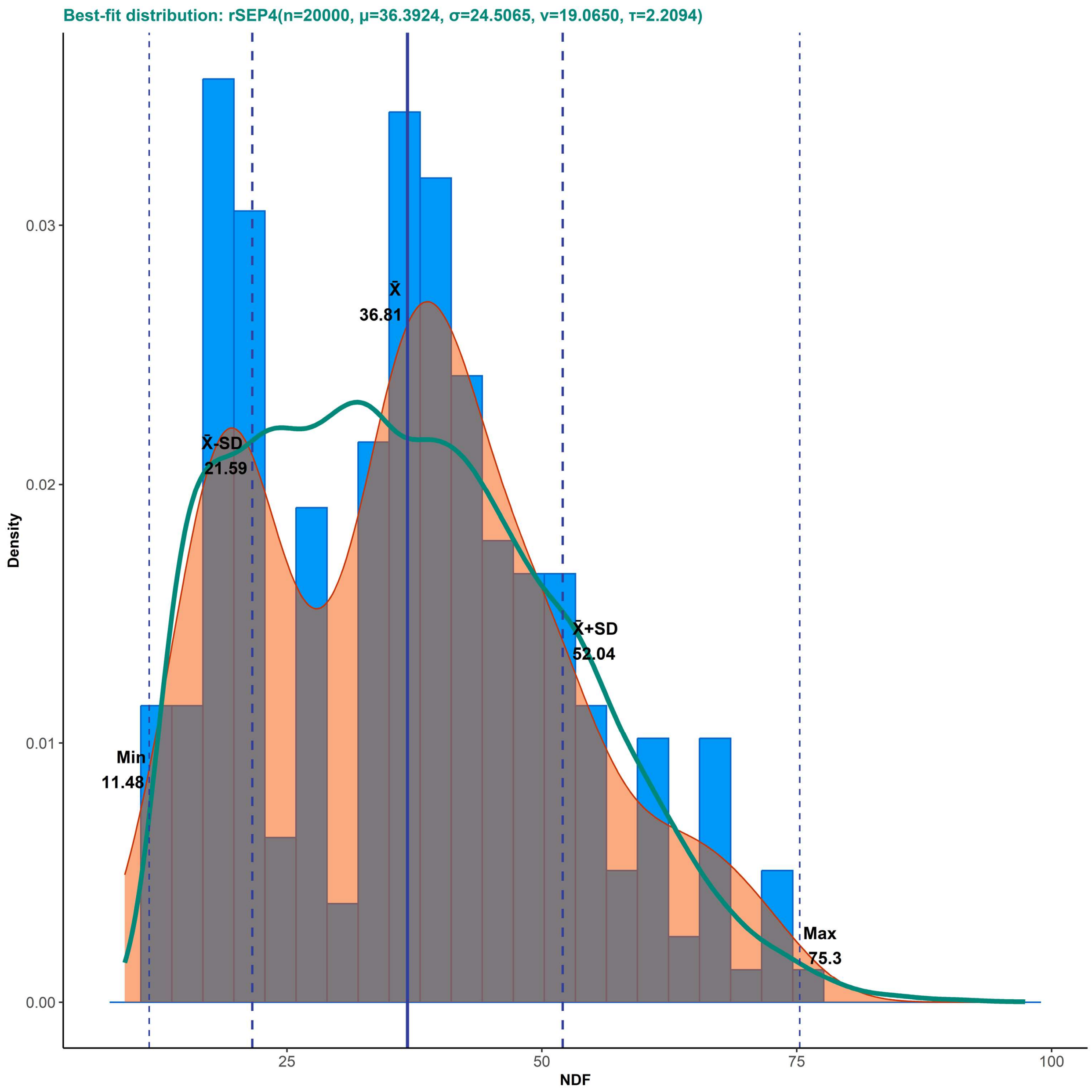

ASAS-NANP SYMPOSIUM: MATHEMATICAL MODELING IN ANIMAL NUTRITION: Synthetic Database Generation for Non-Normal Multivariate Distributions: A Rank-Based Method with Application to Ruminant Methane Emissions (**Supplementary Figure 1**)

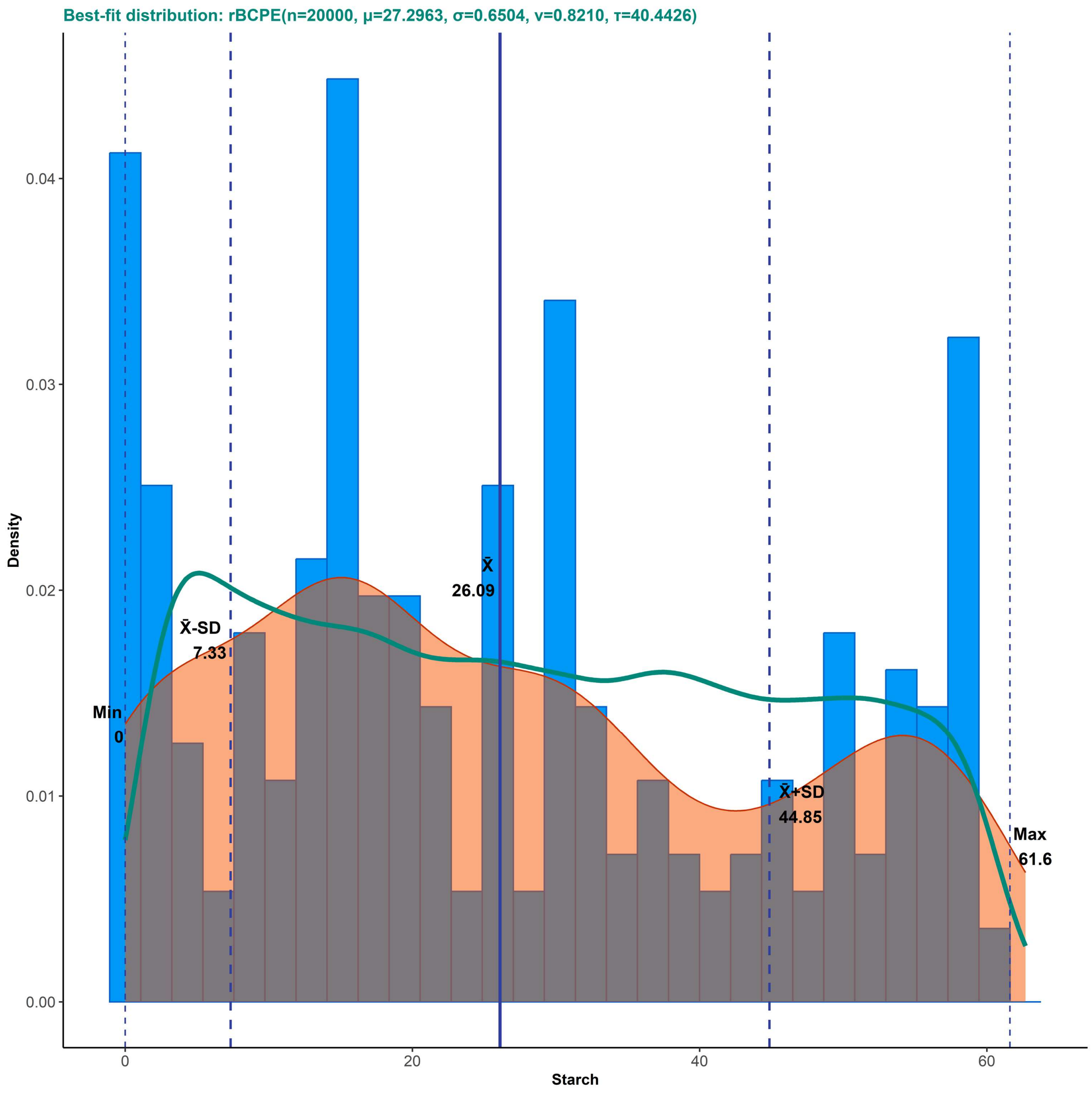

Supplement: skaf136_suppl_Supplementary_Figure_S1 [file skaf136_suppl_supplementary_figure_s1.pdf]
