# Supplementary material for: ASAS-NANP symposium: mathematical modeling in animal nutrition: synthetic database generation for non-normal multivariate distributions: a rank-based method with application to ruminant methane emissions
Source: J Anim Sci. 2025 May 4;103:skaf136. doi: 10.1093/jas/skaf136 (PMC12351256; doi:10.1093/jas/skaf136)
Supplement: skaf136_suppl_Supplementary_Material_S1 [file skaf136_suppl_supplementary_material_s1.pdf]

Supplementary Material 1. R-script code to fit a distribution to the data and generate synthetic data based on the best fit.

```
getDist <- function(var, varname = "", normalonly = FALSE, hist = TRUE, n = 10000,
showtime = F, showtitle = F, ...)
{
  # Function to fit a distribution to the data and generate synthetic data based on
the best fit.
  # If normalonly is TRUE, it fits a normal distribution regardless of the data.
  # If hist is TRUE, it generates a histogram comparing the original and synthetic
data.
  # The number of samples to generate is specified by n.

  require(gamlss)

  # Fit the best distribution
  if (normalonly == FALSE) {
    m1 <- fitDist(var, ...)
  } else {
    m1 <- gamlss(var ~ 1, family = "NO")
  }

  aic <- m1$aic
  pars <- m1$parameters
  lpar <- length(m1$parameters)
  dis <- paste0("r", m1$family[[1]])

  # Generate the synthetic data based on the fitted distribution
  outdis <- switch(lpar,
                   call(dis, n = n, mu = fitted(m1)[1]),
                   call(dis, n = n, mu = fitted(m1)[1], sigma = fitted(m1,
"sigma")[1]),
                   call(dis, n = n, mu = fitted(m1)[1], sigma = fitted(m1,
"sigma")[1], nu = fitted(m1, "nu")[1]),
                   call(dis, n = n, mu = fitted(m1)[1], sigma = fitted(m1,
"sigma")[1], nu = fitted(m1, "nu")[1], tau = fitted(m1, "tau")[1])
  )

  # Create a description of the fitted distribution
  txtdis <- switch(lpar,
                   paste(dis, "(n=", n, ", \u03BC=", formatC(fitted(m1)[1], format =
"f", digits = 4), ")", sep = ""),
                   paste(dis, "(n=", n, ", \u03BC=", formatC(fitted(m1)[1], format =
"f", digits = 4), ", \u03C3=", formatC(fitted(m1, "sigma")[1], format = "f", digits =
4), ")", sep = ""),
```

```
                   paste(dis, "(n=", n, ", \u03BC=", formatC(fitted(m1)[1], format = "f", digits = 4), ", \u03C3=", formatC(fitted(m1, "sigma")[1], format = "f", digits = 4), ", \u03BD=", formatC(fitted(m1, "nu")[1], format = "f", digits = 4), ")", sep = ""),
                   paste(dis, "(n=", n, ", \u03BC=", formatC(fitted(m1)[1], format = "f", digits = 4), ", \u03C3=", formatC(fitted(m1, "sigma")[1], format = "f", digits = 4), ", \u03BD=", formatC(fitted(m1, "nu")[1], format = "f", digits = 4), ", \u03C4=", formatC(fitted(m1, "tau")[1], format = "f", digits = 4), ")", sep = "")
  )

  outval <- try(eval(outdis), silent = TRUE)

  # Fallback to normal distribution if fitting fails
  if (class(outval) == "try-error") {
    mu <- mean(var)
    sd <- sd(var)
    outdis <- paste("rnorm, ", n, ", ", mu, ", ", sd, sep = "")
    subtitle <- paste("Best-fit distribution: ", txtdis, " !FAILED!\n", "Adopted distribution: rnorm(n=", n, ", mu=", formatC(mu, format = "f", digits = 4), ", sigma=", formatC(sd, format = "f", digits = 4), ")", sep = "")
    txtdis <- paste(txtdis, " !FAILED! | rnorm(n=", n, ", mu=", formatC(mu, format = "f", digits = 4), ", sigma=", formatC(sd, format = "f", digits = 4), ")", sep = "")
    outval <- rnorm(n, mu, sd)
    aic <- Inf
  } else {
    subtitle <- paste("Best-fit distribution: ", txtdis, sep = "")
  }

  # Generate histogram and density plots if requested
  if (hist) {
    densMode <- function(x, x_target=0) {
      td <- density(x)
      maxDens <- which.max(td$y)
      density_at_target <- approx(td$x, td$y, xout = x_target)$y
      list(x = td$x[maxDens], y = td$y[maxDens], target = density_at_target)
    }

    d <- as.data.frame(var)
    mean_value <- mean(d$var)
    sd_value <- sd(d$var)
    min_value <- min(d$var)
    max_value <- max(d$var)
    atmode <- densMode(d$var)$y
    atmin <- densMode(d$var,min_value)$target
    atmax <- densMode(d$var,max_value)$target
    at1SDm <- densMode(d$var,mean_value-sd_value)$target
```

```
    at1SDp <- densMode(d$var,mean_value+sd_value)$target
    annotations <- data.frame(
      x = c(round(min_value, 2), round(mean_value, 2), round(max_value, 2),
round(mean_value - sd_value, 2), round(mean_value + sd_value, 2)),
      y = c(atmin, atmode, atmax, at1SDm, at1SDp),
      label = c("Min\n", "\u0058\u0304 \n", "Max\n", "\u0058\u0304-SD \n", "
\u0058\u0304+SD\n")
    )

    # Set title and caption based on the parameters
    title_text <- if (showtitle) paste("Histogram and Density Plots of '", varname,
"'", sep = "") else NULL
    caption_text <- if (showtime) paste(format(Sys.time(), "%A, %d %b %Y, %X"), sep =
"") else NULL

    plot <- ggplot() +
      geom_histogram(data = d, aes(x = var, y = after_stat(density)), color =
"#0066CC", fill = "#0099F8") +
      geom_density(data = d, aes(x = var), color = "#CC3300", fill = "#F85700", alpha
= 0.5) +
      geom_density(data = as.data.frame(outval), aes(x = outval), color = "#00897B",
linewidth = 1.5) +
      geom_text(data = annotations, aes(x = x, y = y, label = paste(label, x)), size
= 4, hjust = c(1.1, 1.1, -0.1, 1.1, -0.1), fontface = "bold") +
      labs(title = title_text, subtitle = subtitle, caption = caption_text, x =
varname, y = "Density") +
      theme_classic() +
      theme(
        plot.title = element_text(color = "#0099F8", size = 16, face = "bold"),
        plot.subtitle = element_text(color = "#00897B", size = 11, face = "bold"),
        axis.title.x = element_text(size = 10, face = "bold"),
        axis.title.y = element_text(size = 10, face = "bold"),
        axis.text.x = element_text(size = 10),
        axis.text.y = element_text(size = 10),
        plot.caption = element_text(face = "italic")
      ) +
      geom_vline(aes(xintercept = mean(var)), color = "#303F9F", linewidth = 1) +
      geom_vline(aes(xintercept = mean(var) + sd(var)), color = "#303F9F", linewidth
= 0.75, linetype = "dashed") +
      geom_vline(aes(xintercept = mean(var) - sd(var)), color = "#303F9F", linewidth
= 0.75, linetype = "dashed") +
      geom_vline(aes(xintercept = min(var)), color = "#303F9F", linewidth = 0.5,
linetype = "dashed") +
      geom_vline(aes(xintercept = max(var)), color = "#303F9F", linewidth = 0.5,
linetype = "dashed")
```

```
    # Save the plot as a TIFF file
    tiff(paste(varname, ".tiff", sep = ""), width = 8000, height = 8000, bg =
"white", res = 800, compress = "lzw")
    print(plot)
    dev.off()
  }

  return(list(dist = txtdis, aic = aic, distval = outval))
}

# Load necessary libraries
library(gamlss)
library(ggplot2)

# Example dataset: Simulate some non-normal data
set.seed(123)
example_data <- rnorm(1000, mean = 50, sd = 10)

# Use the getDist function to fit a distribution and generate synthetic data
result <- getDist(var = example_data, varname = "Example Data", normalonly = FALSE,
hist = TRUE, n = 10000)

# Print the results
print(result$dist)
print(result$aic)

# Display the first few synthetic data values
head(result$distval)
```
