# Supplementary material for: ASAS-NANP symposium: mathematical modeling in animal nutrition: synthetic database generation for non-normal multivariate distributions: a rank-based method with application to ruminant methane emissions
Source: J Anim Sci. 2025 May 4;103:skaf136. doi: 10.1093/jas/skaf136 (PMC12351256; doi:10.1093/jas/skaf136)
Supplement: skaf136_suppl_Supplementary_Material_S2 [file skaf136_suppl_supplementary_material_s2.pdf]

Supplementary Material 2. R-script code for implementing rank-based and copula methods for correlating multiple non-normally distributed variables using Cholesky decomposition.

```
# Function to apply rank-based adjustment
correlate_using_ranks <- function(varmatrix, cormatrix)
{
  require(MASS)

  n <- nrow(varmatrix)
  ncols <- ncol(varmatrix)

  # Generate uncorrelated normally distributed columns
  mu <- rep(0, ncols)
  sigma <- diag(ncols)
  d <- mvrnorm(n, mu, sigma)

  # Correlate the normally distributed columns
  u <- chol(cormatrix)
  z <- d %*% u

  # Rank each column
  rank_z <- apply(z, 2, rank)
  rank_varmatrix <- apply(varmatrix, 2, rank)

  # Allocate original values based on correlated ranks
  z2 <- matrix(NA, n, ncols)
  for (i in 1:ncols) {
    z2[, i] <- varmatrix[match(rank_z[, i], rank_varmatrix[, i]), i]
  }

  return(z2)
}

# Function to apply copula adjustment
correlate_using_copula <- function(varmatrix, cormatrix, iterations = 100, tol = 1e-
5)
{
  library(MASS)
  library(copula)

  n <- nrow(varmatrix)
  ncols <- ncol(varmatrix)

  # Standardize the variables to uniform marginals
  uniform_data <- apply(varmatrix, 2, function(x) rank(x) / (length(x) + 1))
```

```
  # Transform to normal marginals
  normal_data <- qnorm(uniform_data)

  # Initialize correlated data
  correlated_data <- normal_data

  for (iteration in 1:iterations) {
    # Calculate current correlation
    current_corr <- cor(correlated_data)

    # Decompose the desired correlation matrix
    L <- chol(cormatrix)
    current_L <- chol(current_corr)

    # Adjust using Cholesky factors
    adjustment <- correlated_data %*% solve(current_L) %*% L
    correlated_data <- adjustment %*% t(L)

    # Transform back to original marginals
    for (i in 1:ncols) {
      ranks <- rank(correlated_data[, i])
      correlated_data[, i] <- sort(varmatrix[, i])[ranks]
    }

    # Check convergence
    if (iteration %% 5 == 0 && max(abs(cor(correlated_data) - cormatrix)) < tol) {
      break
    }
  }

  return(correlated_data)
}

# Testing
# Generate synthetic non-normal data
set.seed(1983458)
n_samples <- 5000
var1 <- rchisq(n_samples, df = 2)
var2 <- rbeta(n_samples, shape1 = 2, shape2 = 5)
var3 <- rlnorm(n_samples, meanlog = 0, sdlog = 1)
varmatrix <- cbind(var1, var2, var3)

# Create a predefined positive-definite correlation matrix
cormatrix <- matrix(c(
  1, 0.5, 0.3,
```

```
  0.5, 1, 0.4,
  0.3, 0.4, 1
), nrow = 3, ncol = 3)

# Apply the adjustment functions
correlated_data_copula <- correlate_using_copula(varmatrix, cormatrix,500)
correlated_data_ranks <- correlate_using_ranks(varmatrix, cormatrix)

# Verify the correlation matrix
cor(correlated_data_copula)
cor(correlated_data_ranks)
```
