# Supplementary material for: ASAS-NANP symposium: mathematical modeling in animal nutrition: synthetic database generation for non-normal multivariate distributions: a rank-based method with application to ruminant methane emissions
Source: J Anim Sci. 2025 May 4;103:skaf136. doi: 10.1093/jas/skaf136 (PMC12351256; doi:10.1093/jas/skaf136)
Supplement: skaf136_suppl_Supplementary_Material_S3 [file skaf136_suppl_supplementary_material_s3.pdf]

Supplementary Material 3. R-script code for comparing distribution correlation and moments between rank-based and copula methods, using 100 iterations. Replace `[username]` with the appropriate folder name.

```
library(MASS)
library(boot)
library(stats)
library(moments)
library(ggplot2)
library(reshape2)

# Generate a positive-definite correlation matrix
generate_pos_def_corr_matrix <- function(n) {
  matrix <- matrix(rnorm(n^2), n, n)
  corr_matrix <- cov2cor(matrix %*% t(matrix))
  return(corr_matrix)
}

# Calculate moments
calculate_moments <- function(data) {
  mean <- colMeans(data)
  variance <- apply(data, 2, var)
  skewness <- apply(data, 2, skewness)
  kurtosis <- apply(data, 2, kurtosis)
  moments <- data.frame(mean, variance, skewness, kurtosis)
  return(moments)
}

# Reduce sample size for faster computation
n_samples <- 5000
iterations <- 100
n_vars <- 3
corr_diff_values_rank <- list()
corr_diff_values_copula <- list()
original_moments_list <- list()
correlated_moments_rank_list <- list()
correlated_moments_copula_list <- list()

for (i in 1:n_vars) {
  for (j in i:n_vars) {
    corr_diff_values_rank[[paste0("Var", i, "-Var", j)]] <- numeric()
    corr_diff_values_copula[[paste0("Var", i, "-Var", j)]] <- numeric()
  }
}
```

```
for (iter in 1:iterations) {
  cat(paste("Iteration: ",iter,"\n"))
  # Generate synthetic non-normal data
  var1 <- rchisq(n_samples, df = 2)
  var2 <- rbeta(n_samples, shape1 = 2, shape2 = 5)
  var3 <- rlnorm(n_samples, meanlog = 0, sdlog = 1)
  varmatrix <- cbind(var1, var2, var3)

  # Use a predefined positive-definite correlation matrix
  cormatrix <- matrix(c(
    1, 0.5, 0.3,
    0.5, 1, 0.4,
    0.3, 0.4, 1
  ), nrow = 3, ncol = 3)
  # Create a random positive-definite correlation matrix
  #cormatrix <- generate_pos_def_corr_matrix(n_vars)

  # Apply the rank-based function
  correlated_data_rank <- correlate_using_ranks(varmatrix, cormatrix)
  # Apply the copula-based function
  correlated_data_copula <- correlate_using_copula(varmatrix, cormatrix)

  # Calculate the Spearman correlation matrices of the correlated data
  corr_matrix_rank <- cor(correlated_data_rank, method = "spearman")
  corr_matrix_copula <- cor(correlated_data_copula, method = "spearman")

  # Calculate the differences for the rank-based approach
  for (i in 1:n_vars) {
    for (j in i:n_vars) {
      corr_diff_values_rank[[paste0("Var", i, "-Var", j)]] <-
c(corr_diff_values_rank[[paste0("Var", i, "-Var", j)]], cormatrix[i, j] -
corr_matrix_rank[i, j])
    }
  }

  # Calculate the differences for the copula-based approach
  for (i in 1:n_vars) {
    for (j in i:n_vars) {
      corr_diff_values_copula[[paste0("Var", i, "-Var", j)]] <-
c(corr_diff_values_copula[[paste0("Var", i, "-Var", j)]], cormatrix[i, j] -
corr_matrix_copula[i, j])
    }
  }

  # Calculate moments
  original_moments_list[[iter]] <- calculate_moments(varmatrix)
```

```
  correlated_moments_rank_list[[iter]] <- calculate_moments(correlated_data_rank)
  correlated_moments_copula_list[[iter]] <- calculate_moments(correlated_data_copula)
}

# Convert moments lists to data frames
original_moments_df <- do.call(rbind, original_moments_list)
correlated_moments_rank_df <- do.call(rbind, correlated_moments_rank_list)
correlated_moments_copula_df <- do.call(rbind, correlated_moments_copula_list)

# Plot histograms of the differences for the rank-based approach
pdf("c:/users/[username]/desktop/correlation_diff_histograms_rank.pdf", width = 14,
height = 14)
par(mfrow = c(n_vars, n_vars))
for (i in 1:n_vars) {
  for (j in 1:n_vars) {
    if (i <= j) {
      hist(corr_diff_values_rank[[paste0("Var", i, "-Var", j)]], main = paste0("Rank-
based Correlation Difference: Var", i, "-Var", j), xlab = "Difference", xlim = c(-1,
1), breaks = 30)
    }
  }
}
dev.off()

# Plot histograms of the differences for the copula-based approach
pdf("c:/users/[username]/desktop/correlation_diff_histograms_copula.pdf", width = 14,
height = 14)
par(mfrow = c(n_vars, n_vars))
for (i in 1:n_vars) {
  for (j in 1:n_vars) {
    if (i <= j) {
      hist(corr_diff_values_copula[[paste0("Var", i, "-Var", j)]], main =
paste0("Copula-based Correlation Difference: Var", i, "-Var", j), xlab =
"Difference", xlim = c(-1, 1), breaks = 30)
    }
  }
}
dev.off()

# Calculate and print min and max for correlation differences
min_max_corr_diff <- function(corr_diff_list, method) {
  for (i in 1:n_vars) {
    for (j in i:n_vars) {
      diff_values <- corr_diff_list[[paste0("Var", i, "-Var", j)]]
      cat(method, " - Var", i, "-Var", j, " Min: ", min(diff_values), " Max: ",
max(diff_values), "\n")
```

```
    }
  }
}

cat("Rank-based Correlation Differences:\n")
min_max_corr_diff(corr_diff_values_rank, "Rank-based")

cat("\nCopula-based Correlation Differences:\n")
min_max_corr_diff(corr_diff_values_copula, "Copula-based")

# Plot moments
plot_moments <- function(original_moments, correlated_moments, method) {
  original_melt <- melt(original_moments)
  correlated_melt <- melt(correlated_moments)
  original_melt$Method <- "Original"
  correlated_melt$Method <- method

  moments_df <- rbind(original_melt, correlated_melt)

  ggplot(moments_df, aes(x = variable, y = value, fill = Method)) +
    geom_boxplot() +
    facet_wrap(~variable, scales = "free") +
    labs(title = paste(method, "Moments Comparison"), x = "Variable", y = "Value") +
    theme_minimal()
}

pdf("c:/users/[username]/desktop/moments_comparison_rank.pdf", width = 14, height =
10)
print(plot_moments(original_moments_df, correlated_moments_rank_df, "Rank-based"))
dev.off()

pdf("c:/users/[username]/desktop/moments_comparison_copula.pdf", width = 14, height =
10)
print(plot_moments(original_moments_df, correlated_moments_copula_df, "Copula-
based"))
dev.off()
```
