# Supplementary material for: ASAS-NANP symposium: mathematical modeling in animal nutrition: synthetic database generation for non-normal multivariate distributions: a rank-based method with application to ruminant methane emissions
Source: J Anim Sci. 2025 May 4;103:skaf136. doi: 10.1093/jas/skaf136 (PMC12351256; doi:10.1093/jas/skaf136)
Supplement: skaf136_suppl_Supplementary_Table_S1 [file skaf136_suppl_supplementary_table_s1.pdf]

Supplementary Table 1. Descriptive statistics of the original data[1].

ID[2]	BW	DMI	CP	NDF	EE
1	447	7.95±0.305[7.73,8.17]	13.2±1.41[12.2,14.2]	13.3±0.226[13.1,13.4]	3.91±0.0990[3.84,3.98]
2	467±36.1[430,520]	6.29±1.34[4.58,8.27]	14.9±0.274[14.6,15.1]	37.7±12.1[26.6,48.7]	2.92±0.886[2.11,3.73]
3	392±48.3[345,439]	6.32±0.737[5.34,6.94]	14.2±0.787[13.4,15.1]	26.1±11.5[12.7,36.3]	3.33±0.714[2.40,4.01]
4	260	5.94±0.589[5.07,6.38]	14.6	34.7	3.17
5	380	6.84±1.32[5.37,8.31]	16.4±0.327[16.0,16.8]	27.5±5.92[20.3,34.8]	3.50±0.0816[3.40,3.60]
6	400	8.50±0.446[7.85,8.86]	12.7±0.173[12.4,12.8]	32.8±1.16[31.6,34.3]	5.01±1.70[2.46,5.86]
7	255±0.577[254,255]	5.80±0.0513[5.76,5.86]	16.0	45.1	3.45
8	184±21.7[154,210]	3.68±0.575[2.93,4.31]	20.1±5.63[13.8,26.9]	39.3±10.1[25.2,50.5]	4.24±0.288[4.00,4.60]
9	322±5.74[314,327]	5.31±0.170[5.07,5.45]	12.8±1.75[11.5,15.4]	46.2±9.55[35.4,58.5]	2.57±0.163[2.36,2.70]
10	327±1.41[326,328]	3.82±0.594[3.40,4.24]	11.4±2.21[9.88,13.0]	69.4±4.09[66.5,72.3]	1.89±0.0283[1.87,1.91]
11	400	4.60	15.8	30.6	1.88
12	238±18.6[217,258]	4.99±0.437[4.49,5.39]	22.4±5.91[16.1,28.0]	32.1±12.2[21.6,42.7]	4.20±0.283[4.00,4.60]
13	347±16.9[325,365]	5.61±1.76[3.50,7.60]	11.5	36.7	5.20
14	346±10.9[331,357]	4.98±0.585[4.30,5.70]	8.68±0.737[7.80,9.60]	61.1±1.15[59.5,62.1]	3.88±2.04[1.00,5.70]
15	332±4.95[328,335]	4.50±0.0636[4.45,4.54]	14.8	31.2	4.00
16	352±16.3[340,363]	4.80	13.9±0.212[13.7,14.0]	20.9	3.20±0.375[2.93,3.46]
17	482±2.06[480,484]	6.77±0.274[6.43,7.10]	11.8	21.7	3.50
18	614±8.83[608,627]	6.82±3.51[2.76,10.9]	13.8	20.1	2.07
19	286]	3.84	17.5	50.0	2.55
20	513±12.2[495,526]	8.11±0.474[7.54,8.65]	12.5±0.312[12.0,12.8]	34.2±5.97[28.0,40.6]	3.23±0.327[2.90,3.73]
21	357±4.73[352,361]	4.27±0.0613[4.20,4.32]	20.0±3.19[16.4,22.4]	37.5±1.98[35.7,39.6]	10.0±6.65[2.80,15.9]

ID[2]	BW	DMI	CP	NDF	EE
22	218	5.20±0.0862[5.10,5.31]	15.2±2.39[13.1,17.4]	15.3±3.08[11.5,18.8]	6.46±0.240[6.25,6.68]
23	324±4.65[320,330]	6.23±0.299[5.80,6.50]	16.5±3.27[13.3,20.2]	17.1±1.95[14.5,18.7]	6.85±1.21[5.80,8.30]
24	362	7.38±0.253[7.09,7.71]	18.5±0.377[18.1,19.0]	17.0±2.69[14.4,20.3]	5.50±0.294[5.20,5.90]
25	503	7.74±0.579[6.88,8.11]	13.3±0.940[12.7,14.7]	12.1±0.446[11.6,12.5]	3.37±0.263[3.06,3.67]
26	464	7.80±0.581[7.29,8.61]	20.1±2.43[17.8,22.4]	19.4±2.31[17.1,21.4]	6.87±0.839[6.12,7.70]
27	479±7.83[471,489]	7.11±0.198[6.90,7.32]	15.0±0.0350[15.0,15.1]	13.4±0.302[13.2,13.9]	6.26±2.53[3.00,8.71]
28	407±47.1[361,454]	7.93±0.947[7.03,9.46]	15.4±1.49[14.4,18.1]	33.1±4.07[26.7,38.5]	4.43±0.810[3.56,5.53]
29	279±12.0[270,287]	6.05±0.919[5.40,6.70]	11.4±1.91[10.0,12.7]	50.6±2.26[49.0,52.2]	3.95±1.91[2.60,5.30]
30	493±41.0[448,556]	6.52±1.28[5.30,8.22]	8.63	66.8	2.34
31	290	5.28±1.52[3.64,7.77]	18.7±0.765[17.5,19.3]	45.5±3.74[42.0,49.0]	2.65±0.267[2.40,2.90]
32	388	8.54±0.384[8.13,9.05]	19.3±4.66[13.0,23.5]	34.6±2.70[32.5,38.5]	4.43±1.28[3.00,5.60]
33	529	9.39±0.593[8.76,10.2]	19.3±4.93[12.2,23.1]	24.1±3.50[19.4,27.9]	3.90±1.63[2.00,5.40]
34	326±74.8[261,391]	4.24±2.25[2.29,6.23]	16.7±3.29[13.8,19.5]	19.6±0.231[19.4,19.8]	4.05±0.635[3.50,4.60]
35	176±0.707[175,176]	3.98±0.0919[3.91,4.04]	15.6	39.9	4.76
36	461±114[315,575]	7.37±0.726[6.64,8.27]	13.7±0.122[13.5,13.8]	53.8±0.917[52.7,54.8]	4.34
37	459	7.26±0.971[5.93,8.55]	15.1±0.825[13.9,16.2]	44.3±6.83[34.5,53.0]	2.43±0.164[2.27,2.70]
38	140±7.21[134,148]	2.83±0.153[2.74,3.01]	9.77±0.115[9.70,9.90]	54.0±9.01[45.2,63.2]	5.90
39	366±13.6[353,380]	5.99±2.09[3.58,7.31]	10.4±8.78[2.40,19.8]	55.7±31.0[20.0,75.3]	1.67±0.586[1.00,2.10]
40	526±1.55[525,528]	8.63±0.171[8.40,8.80]	12.5±0.0577[12.4,12.5]	38.3±0.330[37.9,38.6]	3.46±0.0299[3.42,3.49]
41	312	7.28±0.342[6.79,7.71]	14.5	36.2	3.73±1.51[3.20,7.46]
42	141	4.15±0.318[3.92,4.37]	12.9	47.3	5.60
43	599	6.28±0.0954[6.19,6.38]	8.87±0.153[8.70,9.00]	45.3±3.48[42.6,49.2]	2.83±0.153[2.70,3.00]
44	508±17.9[495,528]	8.67±0.0208[8.65,8.68]	13.3	19.9	4.64

ID[2]	BW	DMI	CP	NDF	EE
45	180±12.6[161,193]	3.56±0.670[2.70,4.33]	10.5±0.272[10.1,10.9]	53.6±2.72[50.5,56.8]	2.14±0.463[1.61,2.82]
46	604±34.3[551,662]	9.70±2.07[7.60,12.2]	18.5±0.920[17.4,19.6]	44.3±1.39[41.7,45.3]	2.08±0.103[1.90,2.20]
47	321±11.7[308,335]	5.66±1.85[3.60,7.78]	17.1±0.535[16.6,17.7]	31.5±9.87[20.6,41.3]	2.48±0.419[2.20,3.10]
48	549	8.50	11.7	37.6	2.70
49	637	7.15	11.3	38.6	1.85
50	325	4.07	10.9	18.8	4.39
51	213±1.41[212,214]	4.65±0.205[4.50,4.79]	25.7±2.23[24.1,27.2]	17.7±1.62[16.6,18.9]	2.29±0.0141[2.28,2.30]
52	294±27.9[269,324]	4.63±1.03[3.50,5.50]	10.6	36.3	3.50
53	375	4.97±0.751[4.10,5.40]	13.1±1.14[12.3,14.4]	43.8±18.3[24.3,60.5]	4.59±0.695[4.03,5.37]
54	722±12.0[713,730]	10.5±0.283[10.3,10.7]	14.5±0.141[14.4,14.6]	28.5±0.636[28.0,28.9]	3.96±1.75[2.72,5.20]
55	269±6.84[256,276]	4.59±0.150[4.33,4.77]	18.2±4.11[11.8,24.4]	59.2±12.7[39.6,73.1]	4.05±0.818[2.97,5.51]
56	305±10.1[291,315]	5.50±1.00[4.60,6.60]	21.9±2.66[19.6,24.2]	49.5±14.2[37.2,61.8]	5.80±0.346[5.50,6.10]
57	600	8.64±0.640[7.78,9.30]	12.1	40.7	3.00
58	477±9.84[462,482]	7.60±0.796[6.50,8.40]	13.1	38.2	3.60
59	372	7.28±1.01[6.23,8.70]	13.9±0.657[13.3,14.5]	34.4±8.05[27.0,41.7]	3.42±0.361[3.09,3.75]
60	397±90.1[319,475]	6.96±0.704[6.09,7.55]	13.0±0.866[12.2,13.7]	27.8±9.93[19.2,36.4]	2.79±0.606[2.26,3.31]
61	334±3.32[330,338]	6.86±0.405[6.31,7.29]	18.8±3.75[15.5,22.0]	41.4±0.523[40.9,41.9]	3.71±1.33[2.55,4.88]
62	452±4.24[449,455]	3.47	13.8	15.7	6.32
63	275±1.60[273,276]	5.10	9.93±1.17[7.88,11.1]	56.6±6.22[50.5,68.1]	2.70±0.470[1.94,3.18]

[1] Values represent average ± standard deviation [minimum, maximum].

[2] 1=Archibeque et al. (2006), 2=Baber et al. (2020), 3=Beauchemin and McGinn (2005), 4=Beauchemin and McGinn (2006b), 5=Beauchemin and McGinn (2006a), 6=Beauchemin et al. (2007b), 7=(Beauchemin et al., 2007a), 8=Beever et al. (1985), 9=Beever et al. (1988), 10=Birkelo et al. (1986), 11=Boadi et al. (2001), 12=Cammell et al. (1986), 13=Chaokaur et al. (2015), 14=Chuntrakort et al. (2014), 15=Cole and McCroskey (1975), 16=Croka and Wagner (1975), 17=Crossland et al. (2018), 18=Delfino et al. (1988), 19=Derno et al. (2005), 20=Fuller et

al. (2020), 21=Haaland et al. (1981), 22=Hales et al. (2012), 23=Hales et al. (2013),24=Hales et al. (2014), 25=Hales et al. (2015a), 26=Hales et al. (2015b), 27=Hales et al. (2017), 28=Hammond et al. (2015), 29=Hellwing et al. (2012), 30=Hemphill et al. (2018), 31=Hironaka et al. (1996), 32=Hünerberg et al. (2013b), 33=Hünerberg et al. (2013a), 34=Jennings et al. (2018), 35=Jiao et al. (2013), 36=Jiao et al. (2015), 37=Kirkpatrick et al. (1997), 38=Kongphitee et al. (2018), 39=Kurihara et al. (1999), 40=Lee et al. (2015), 41=McGinn et al. (2004), 42=Mohammed et al. (2004), 43=Nishida et al. (2007), 44=Nkrumah et al. (2006), 45=Ortigues et al. (1990), 46=Reynolds and Tyrrell (2000), 47=Reynolds et al. (1991), 48=Romero-Perez et al. (2014), 49=Romero-Perez et al. (2015), 50=Rumpler et al. (1986), 51=Shreck et al. (2017), 52=Tangjitwattanachai et al. (2015), 53=Thornton and Owens (1981), 54=Troy et al. (2015), 55=Tyrrell et al. (1992), 56=Varga et al. (1990), 57=Vyas et al. (2014), 58=Vyas et al. (2016b), 59=Vyas et al. (2016a), 60=Vyas et al. (2018), 61=Waldo et al. (1997), 62=Walter et al. (2016), and 63=Wei et al. (2018)

Supplementary Table 1. Descriptive statistics of the original data[1] (continued).

ID[2]	Starch	CH_4	OM	ADF
1	64.4±2.54[62.6,66.2]	126±20.5[111,140]	89.3	6.11±0.127[6.02,6.20]
2	30.9±25.6[7.49,54.3]	121±29.5[83.3,167]	---	---
3	40.9±14.3[27.3,58.3]	111±49.1[62.1,171]	94.0±0.913[92.7,94.8]	10.2±6.40[3.50,16.3]
4	14.5	150±28.6[108,171]	91.0	21.1
5	30.4±12.6[14.9,45.8]	142±23.8[114,169]	93.3±0.776[92.3,94.2]	16.7±4.16[11.6,21.8]
6	27.0	151±23.7[120,177]	91.6±0.938[90.8,92.6]	19.1±1.45[17.5,21.0]
7	15.0	99.2±0.503[98.7,99.7]	91.4	26.1
8	0	93.5±16.1[66.3,108]	---	---
9	13.4±17.2[0,35.8]	135±13.6[117,150]	---	---
10	0	75.5±17.2[63.4,87.7]	---	---
11	20.3]	93.7	91.3	21.5
12	0	80.1±9.09[70.0,90.4]	---	---
13	30.3	145±21.2[121,168]	---	---
14	14.9±4.30[9.99,20.0]	110±48.2[65.8,170]	---	---
15	50.0	67.3±13.9[57.5,77.1]	95.3	15.4
16	56.0	56.5±8.72[50.4,62.7]	---	---
17	49.2	50.5±4.06[45.8,55.1]	---	---
18	31.0	158±63.2[86.1,240]	90.8	9.81
19	0	100	---	---
20	30.7±9.94[21.1,45.3]	140±30.8[104,174]	---	---
21	11.3±13.6[3.10,27.0]	82.1±12.1[71.3,95.2]	---	---

ID[2]	Starch	CH_4	OM	ADF
22	46.1±4.22[41.3,50.9]	47.0±3.91[42.6,52.1]	---	---
23	50.3±11.0[39.1,60.7]	59.0±10.6[51.1,73.6]	---	---
24	50.5±3.54[46.4,54.6]	93.5±10.2[80.3,103]	---	---
25	55.9±4.91[50.2,61.6]	108±5.23[103,115]	---	---
26	35.5±11.5[21.4,46.5]	84.9±7.03[78.8,94.7]	---	---
27	53.9±2.36[51.5,56.9]	67.6±12.3[55.3,80.3]	---	---
28	21.1±8.20[10.0,32.9]	200±11.0[184,220]	95.6±0.849[94.7,96.8]	22.5±3.44[17.1,26.8]
29	10.3±0.354[10.0,10.5]	135±24.0[118,152]	93.4±0.566[93.0,93.8]	---
30	4.11	122±8.63[114,133]	---	---
31	2.00	212±31.6[184,261]	88.0	35.0±4.28[31.0,39.0]
32	22.0±9.23[16.8,35.8]	194±23.6[174,228]	91.8±0.350[91.6,92.3]	22.1±2.73[18.0,23.7]
33	38.7±10.9[31.9,55.0]	131±11.6[119,144]	94.8±0.842[94.0,95.6]	12.0±3.20[7.30,14.2]
34	47.4±2.94[44.8,49.9]	64.3±16.8[49.0,81.7]	---	---
35	21.6	93.6±4.38[90.5,96.7]	---	---
36	7.86	175±14.2[159,194]	---	---
37	24.9±15.3[0,42.8]	174±40.4[116,226]	---	---
38	20.3±11.5[8.83,31.8]	53.1±4.18[50.7,57.9]	---	---
39	13.5±23.3[0,40.4]	135±66.0[71.3,203]	---	---
40	39.6±0.369[39.2,40.1]	167±17.0[145,183]	---	---
41	14.5	165±16.3[129,181]	91.9	16.9
42	16.7	81.2±18.9[67.8,94.5]	90.8	28.1
43	32.6	148±6.83[142,155]	94.5±0.379[94.1,94.8]	27.2±3.79[24.5,31.5]
44	57.2	122±19.9[99.2,136]	---	---

ID[2]	Starch	CH_4	OM	ADF
45	14.2±0.946[13.1,15.4]	88.0±12.7[74.7,101]	---	---
46	15.9±2.28[13.9,19.6]	213±46.2[159,268]	90.4±0.892[89.2,91.8]	26.7±1.19[25.2,27.8]
47	34.9±17.3[19.9,49.8]	104±32.5[65.0,142]	---	---
48	31.8	207	92.9	20.6
49	33.8	158	92.5	22.4
50	57.6	83.3±5.68[72.1,89.4]	96.7	9.20
51	6.18±8.74[0,12.4]	86.1±8.17[80.4,91.9]	---	---
52	30.9	131±20.4[109,148]	---	---
53	29.1±17.5[11.4,46.4]	155±21.9[133,177]	91.1±1.46[89.8,92.7]	26.1±14.0[11.5,39.5]
54	27.6±1.91[26.2,28.9]	249±4.24[246,252]	94.7±0.0707[94.6,94.7]	19.5±0.141[19.4,19.6]
55	5.24±4.16[0.800,11.0]	96.8±9.18[78.6,110]	91.9±1.28[90.2,93.6]	40.7±4.14[34.3,46.3]
56	0	127±20.0[105,145]	---	---
57	18.4	188±15.9[172,210]	90.5	21.3
58	22.5	178±10.1[167,190]	92.9	24.8
59	22.4±9.50[13.7,31.0]	133±15.3[111,154]	94.6±1.70[93.0,96.1]	13.2±6.52[7.20,19.1]
60	41.9±17.5[26.7,57.0]	135±33.3[102,176]	94.3±1.27[93.2,95.4]	15.4±8.08[8.40,22.4]
61	14.6±13.4[3.00,26.2]	168±11.8[158,182]	93.5±1.36[92.3,94.7]	27.5±4.25[23.8,31.5]
62	53.3	28.6±5.03[25.0,32.2]	---	---
63	28.0±9.16[14.9,37.4]	108±9.49[92.7,118]	---	---

[1] Values represent average ± standard deviation [minimum, maximum].

[2] 1=Archibeque et al. (2006), 2=Baber et al. (2020), 3=Beauchemin and McGinn (2005), 4=Beauchemin and McGinn (2006b), 5=Beauchemin and McGinn (2006a), 6=Beauchemin et al. (2007b), 7=(Beauchemin et al., 2007a), 8=Beever et al. (1985), 9=Beever et al. (1988), 10=Birkelo et al. (1986), 11=Boadi et al. (2001), 12=Cammell et al. (1986), 13=Chaokaur et al. (2015), 14=Chuntrakort et al. (2014), 15=Cole and McCroskey (1975), 16=Croka and Wagner (1975), 17=Crossland et al. (2018), 18=Delfino et al. (1988), 19=Derno et al. (2005), 20=Fuller et

al. (2020), 21=Haaland et al. (1981), 22=Hales et al. (2012), 23=Hales et al. (2013),24=Hales et al. (2014), 25=Hales et al. (2015a), 26=Hales et al. (2015b), 27=Hales et al. (2017), 28=Hammond et al. (2015), 29=Hellwing et al. (2012), 30=Hemphill et al. (2018), 31=Hironaka et al. (1996), 32=Hünerberg et al. (2013b), 33=Hünerberg et al. (2013a), 34=Jennings et al. (2018), 35=Jiao et al. (2013), 36=Jiao et al. (2015), 37=Kirkpatrick et al. (1997), 38=Kongphitee et al. (2018), 39=Kurihara et al. (1999), 40=Lee et al. (2015), 41=McGinn et al. (2004), 42=Mohammed et al. (2004), 43=Nishida et al. (2007), 44=Nkrumah et al. (2006), 45=Ortigues et al. (1990), 46=Reynolds and Tyrrell (2000), 47=Reynolds et al. (1991), 48=Romero-Perez et al. (2014), 49=Romero-Perez et al. (2015), 50=Rumpler et al. (1986), 51=Shreck et al. (2017), 52=Tangjitwattanachai et al. (2015), 53=Thornton and Owens (1981), 54=Troy et al. (2015), 55=Tyrrell et al. (1992), 56=Varga et al. (1990), 57=Vyas et al. (2014), 58=Vyas et al. (2016b), 59=Vyas et al. (2016a), 60=Vyas et al. (2018), 61=Waldo et al. (1997), 62=Walter et al. (2016), and 63=Wei et al. (2018)
